# Bursting Reverberation as a Multiscale Neuronal Network Process Driven by Synaptic Depression-Facilitation

**DOI:** 10.1371/journal.pone.0124694

**Published:** 2015-05-27

**Authors:** K. Dao Duc, C.Y. Lee, Pierre Parutto, Dror Cohen, Menahem Segal, Nathalie Rouach, David Holcman

**Affiliations:** 1 Ecole Normale Supérieure, IBENS 46 rue d’Ulm 75005 Paris, France; 2 Neuroglial Interactions in Cerebral Physiopathology; Center for Interdisciplinary Research in Biology; CNRS UMR 7241; INSERM U1050; College de France; Paris, France; 3 Department of Neurobiology, Weizmann Institute 76100 Rehovot Israel; Georgia State University, UNITED STATES

## Abstract

Neuronal networks can generate complex patterns of activity that depend on membrane properties of individual neurons as well as on functional synapses. To decipher the impact of synaptic properties and connectivity on neuronal network behavior, we investigate the responses of neuronal ensembles from small (5–30 cells in a restricted sphere) and large (acute hippocampal slice) networks to single electrical stimulation: in both cases, a single stimulus generated a synchronous long-lasting bursting activity. While an initial spike triggered a reverberating network activity that lasted 2–5 seconds for small networks, we found here that it lasted only up to 300 milliseconds in slices. To explain this phenomena present at different scales, we generalize the depression-facilitation model and extracted the network time constants. The model predicts that the reverberation time has a bell shaped relation with the synaptic density, revealing that the bursting time cannot exceed a maximum value. Furthermore, before reaching its maximum, the reverberation time increases sub-linearly with the synaptic density of the network. We conclude that synaptic dynamics and connectivity shape the mean burst duration, a property present at various scales of the networks. Thus bursting reverberation is a property of sufficiently connected neural networks, and can be generated by collective depression and facilitation of underlying functional synapses.

## Introduction

Synchronous neuronal activity is determined by intrinsic and synaptic properties of neurons participating in the network. Patterned stimulation of one of the participating neurons can lead to reverberations of selective neural elements [1, 2]. The cellular parameters that determine the properties of a synchronized network burst are still not fully understood [3, 4]. Earlier studies proposed that membrane currents generated by calcium and calcium-gated potassium channels create plateau potentials which can depolarize neurons for seconds [5, 7]. On the other hand, synaptic properties are essential for the generation and maintenance of the bursts [6, 8 9]. Recent studies propose that specific ‘hub’ long range GABAergic neurons in the hippocampus are the ones to trigger and synchronize network bursts [10]. In hippocampal neurons grown in microcultures [9], network bursts induced by a single action potential triggered in one neuron of the network, reverberate for several seconds. This reverberating burst is followed by a long refractory period, assumed to be caused by the depletion of neurotransmitters from presynaptic terminals [9].

In the present study, we investigated this property across networks of various scales. Neuronal network modeling has shown that facilitation-depression networks can underlie reverberation in large neuronal ensembles [11, 12, 13, 15], suggesting that a few seconds of reverberation correspond to a burst duration that in another context characterizes short-term memory [15]. Mean-field modeling approaches are constantly used to study the effect of synaptic connection on network properties [11, 16, 17].

Because the mean-field description of depression-facilitation synapses does not depend on the number of neurons, small and large neuronal ensembles with similar local connectivity should produce similar responses. Facilitation and depression are short-term neuronal properties, detected in electrophysiological experiments as changes in the amplitude of excitatory postsynaptic currents (EPSCs) evoked by a paired stimulation with short inter-stimulus intervals (tens of milliseconds) [11]. Both mechanisms originate at the synapse and while depression is associated with depletion of presynaptic vesicles, facilitation reflects an increased vesicular release probability due to accumulation of residual calcium at the presynaptic terminal. These processes cause modulation of synaptic transmission in the range of milliseconds to several seconds [18, 19]. From the analysis of the model, we report here collective depression and facilitation that characterizes the ensemble behavior of many synapses, leading to much longer time scales than the ones associated with single neuronal of synaptic unit responses.

We investigated the behavior of small neuronal networks (2–20 neurons) growing on permissive islands. A single intracellular stimulus evoked a burst epoch marked by synchronous activity lasting several seconds (reverberation period) that depends on the local synaptic properties. The depression-facilitation model predicts that the burst duration has a maximum as a function of the synaptic connectivity (the other synaptic properties are constant) and we also investigate the effect of changing the main parameters of the model. In addition, the depression-facilitation model shows that the facilitation variable mediated in part by calcium dynamics defines the duration of the first evoked burst, but not of a second one induced 5 seconds later. Although the model contains eight variables, five are already fixed from the literature and three will be extracted from experimental data. The model reproduces the time course of the recorded electrophysiological responses. Finally, we show that bursting reverberation is a general property that can be found in more organized neuronal ensembles such as in pyramidal cells from acute hippocampal slices. However, the reverberation duration is much shorter compared to the one we report in neuronal cultures.

We conclude that bursting reverberation is a feature that depends on intrinsic properties of depression-facilitation synaptic ensembles, which persists in both small and large neuronal network: Bursting reverberation results from synaptic properties and overall network connectivity. Finally, depression-facilitation properties define the duration of the synchronous bursting activity.

## Results

**Fig 1:**
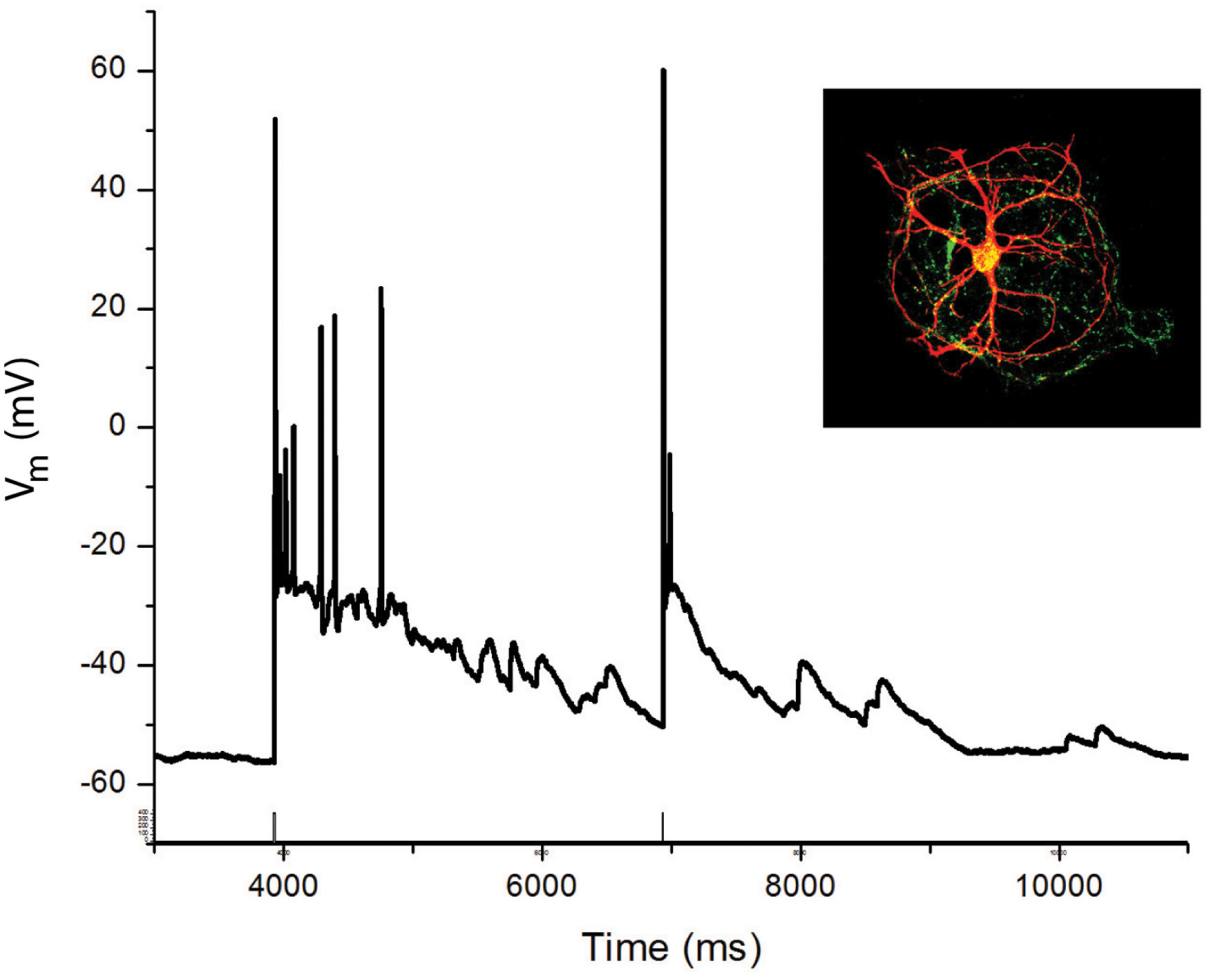
Bursting reverberation in small neuronal islands. A illustration of a network island where neurons are recorded, and filled with biocytin for immunohistochemical identification (red). The island was co-stained with synaptophysin (green dots). B Whole cell recording from a neuron in the island, illustrating responses to two short current pulses that evoke action potentials in the recorded neuron. The first current stimulation evoked an initial spike followed by a depolarization and several additional spikes. The second current pulse, applied 2.5 seconds later produced only a single spike, that was followed by a smaller reverberating network burst, as seen in response to the first stimulus. Scale bar: 20 mV, 1 s.

**Fig 2:**
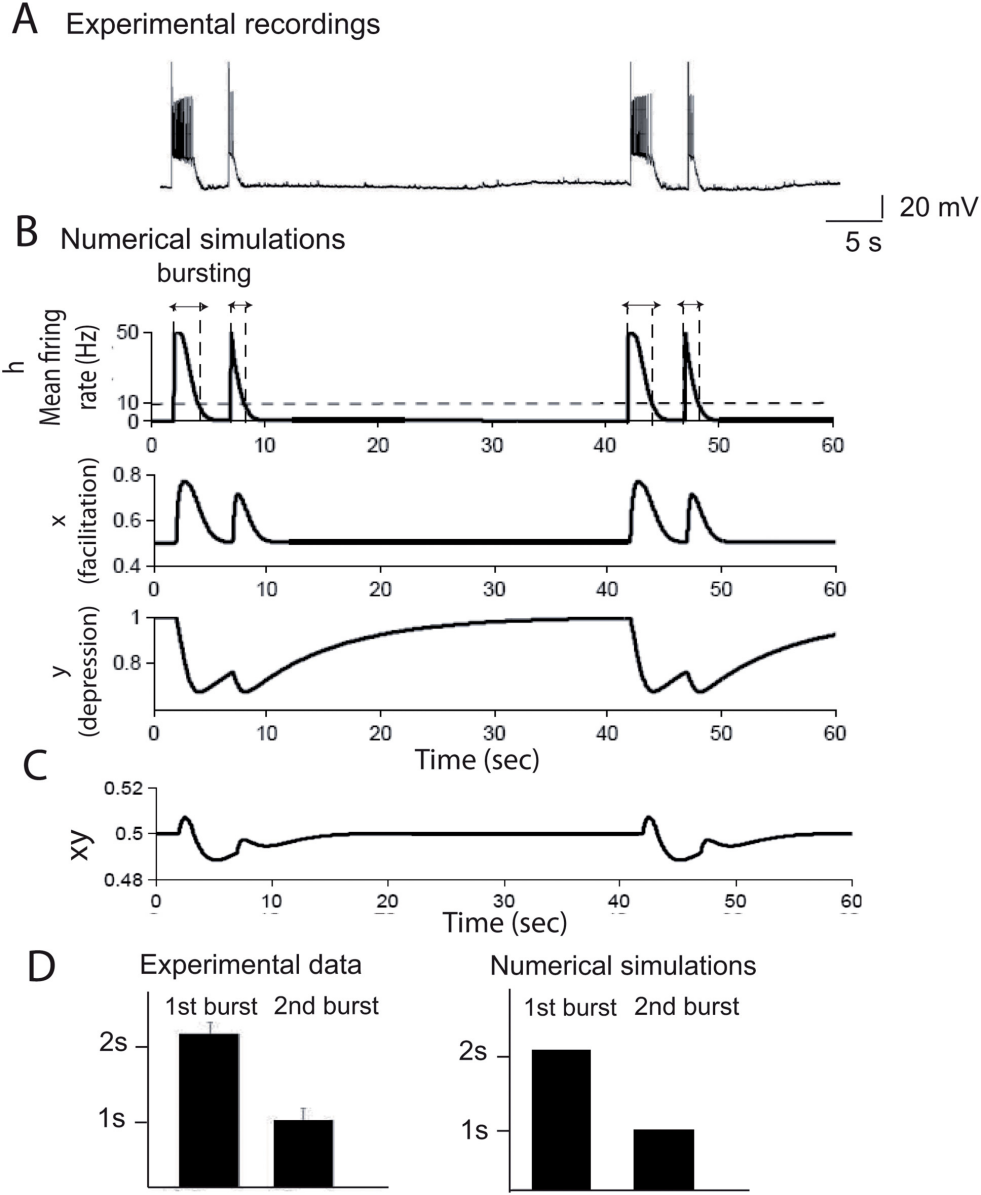
The synaptic depression-facilitation model accounts for bursting reverberation. *(A)* Evoked bursts generated in a microculture hippocampal neurons, with a single action potential evoked alternately at 5 and 35 seconds intervals, showing a reduction in burst duration when the bursts are generated at the short interval. *(B)* Following the experimental protocol, we simulated with Eq 1 and parameters of Table 1 the mean firing rate response, plotted as a function of time. The corresponding facilitation and depression variables show the level of neuronal activity underlying the overall dynamics. *(C)* Plot of the product *xy*, which accounts for the total synaptic modulation, as a function of time. *(D)* Burst durations at 35 seconds and 5 seconds intervals. We compare the burst durations from experimental data (n = 20) with numerical simulations of the model Eq 1.

To study the dynamics of neuronal networks and extract the fundamental properties underlying the network activity, we use two types of experiments with a mean-field neural network modeling based on depression-facilitation. The first experiments involve hippocampal neurons in culture, forming a small group of neurons (between 5–30) (Fig 1). In such neuronal ensemble, an action potential evoked at any neuron was able to generate a burst lasting 1–4 seconds. Furthermore, when two action potentials were evoked at 5 seconds interval, the duration of the second burst was markedly reduced compared to the first one, while no change was found when the intervals between the bursts was longer than 35 seconds (Fig 2A) [21]. Synaptic vesicles were required to sustain bursting, and calcium was determinant in defining the duration of the first burst [9]. In the second experimental setting, we studied reverberation in hippocampal pyramidal cells from acute brain slices.

### 0.1 Reverberation is present in a generalized synaptic depression-facilitation model

Our goal here is to investigate whether neuronal network bursts can originate from synaptic properties, since neuronal intrinsic properties have recently been suggested not to play an important role [9]. Thus we investigated whether the depression-facilitation property of synapses can generate long lasting bursts. We adopt here a general mean-field model, to describe sufficiently connected and homogeneous neural network, where the synaptic dynamics of a single homogeneous excitatory neuronal population is described by the firing rate *h*, a facilitation parameter *x* and the running fraction of neurotransmitter available (depression) *y*[11] see also [20]. Although the depression variable is usually associated with a decay in the vesicular release probability, the facilitation variable has been suggested to depend on presynaptic calcium dynamics. When a short stimulation is generated at a time *t*_*stim*_, the overall dynamics is described by the generalized equations

$$\begin{array}{ccc} \tau \dot{h} & = & -h+Jxyh^{+}+\tau H\delta \left(t-t_{stim}\right)\\ \dot{x} & = & \frac{X-x}{t_{f}}+K\left(1-x\right)h^{+}\\ \dot{y} & = & \frac{1-y}{t_{r}}-Lxyh^{+}, \end{array}$$ (1)

where an experimental population spike stimulation is accounted for in our equation by the term *Hδ*(*t* − *t*_*stim*_), which sets at time *t*_*stim*_ the average firing rate of the network to the value *H* (≈ 50 Hz). The model of Eq 1 does not account for neuronal inhibition, which could have be added using an equation for the inhibitory neuronal voltage. However, depression prevents the system from generating epileptic type behavior and in the experimental data we analyzed here, inhibitory neurons were suppressed pharmacologically. Thus we have not taken into account inhibition. The average population firing rate is given by *R*(*h*) = *h* ^+^ = *max*(*h*,0), which is a threshold linear function of synaptic current [13]. The term *Jxy* reflects the combined effect of synaptic short-term dynamics and network activity. The second equation describes the facilitation dynamics, which enters into a depression process described in the third equation [11]. *J* measures the mean number of connections (synapses) per neurons, as derived analytically in [14]. We have here distinguished *K* and *L* which describe how the firing rate is transformed into molecular events that are changing the duration and the probability for vesicular release respectively. *X* is the steady state of the facilitation variable *x*. Thus Eq 1 generalize the classical depression-facilitation model. More precisely, in a biophysical context, *L* is the rate at which vesicles are depleted for a given firing rate and K measures the rate of facilitation. This model extends the classical equations presented in [15]. The two time scales *t*_*f*_ and *t*_*r*_ define the recovery of a synapse from the network activity. Although, there are 8 parameters in this model: 5 are almost the same as the one published in [15, 13, 22]. We will be left with three parameters to identify, which is a significant reduction of the phase space. In that context, we decided to see whether or not by tuning the three remaining parameters: the two time scales *t*_*f*_ and *t*_*r*_ and the functional network connectivity J, we could reproduce the phenomenology of bursting. Indeed, the initial long phase could be described by facilitation, while the depression will force the burst to terminate. Finally, we note that the present mean-field approach does not account for single spikes generated by a single cell, but only for elicited spikes that are recruiting the ensemble of the neuronal network. Indeed, not all electrical stimulations lead to a burst. Thus the model starts at a population spike level, when a single spike starting in a single neuron was successful in initiating sufficient spikes in all other connected neurons, leading to a bursting activity.

In that context, we decided to see whether by tuning the two time scales *t*_*f*_ and *t*_*r*_ and the network connectivity J, we could reproduce the phenomenology of bursting. Indeed, the initial long phase could be described by facilitation, while the depression will force the burst to terminate.

Following the experimental protocols described above (see also Material and Methods), we explore how the synaptic-depression model could reproduce the two bursts at 5 and 35 seconds intervals. We simulated such dynamics using Eq 1 and identified the corresponding parameters (J and *t*_*f*_ are given in Table 1): the stimuli activates the network which generates a similar behavior as observed in the experimental data, i.e. a reduction in the second burst duration when it was generated at a short interval (5 seconds) after a priming burst (Fig 2B–2D and table A in S1 File). We show in figure Fig. A in S1 File, the effect of changing the bursting interval: after 10 seconds, the ratio returns to one. In agreement with the experimental result, the model shows no changes in the burst duration in the second burst when evoked after 35 seconds. To examine what determines the slow refractoriness of network bursts, we plotted in Fig 2B the depression and facilitation parameters and found that facilitation allows the initial spike evoked in a single neuron to activate the full network, which then disappears at a time scale of *t*_*f*_ = 1.3 s, while the slow depression component prevented the neuronal ensemble from reactivation, when a second burst is generated at a short interval. We took 1.3 s for the decay time constant of facilitation [11], which is larger than the decay of calcium, reported to be order of the order of 200 ms [24]. This difference shows that the synaptic facilitation time scale we use is not exactly following the calcium dynamics.

**Table 1:** Model parameters.

Parameter	Islands	Acute slices
*τ*	0.01s [22]	0.01 s
*t*_*f*_	1.3s (compared to 1.5s in [22])	1.3 s
*t*_*r*_	2 s	20 s
*J*	1.98	2.06
*K*	0.004Hz [15]	0.004 Hz
*L*	0.0054 Hz (compared to 0.005 in [13])	0.037 Hz
*X*	0.5 [15]	0.5
*H*	50 Hz [15]	50 Hz

Using the combination of the modeling approach with the electrophysiological recording, we conclude that the long recovery in the paired pulse network protocol is induced by the network synaptic depression. Although depression decay with a single exponential time scale of *t*_*r*_ = 2 s, it could last for tens of seconds. This decay is compatible with the depletion of synaptic vesicles, which induces depression. Indeed during vesicle fusion, the readily releasable pool is depleted with a time scale of less than 100 ms, while long stimulations can activate other pools, which can take seconds to recover [23]. In addition, the review [23] suggests that the long lasting bursting (2 seconds), which was shown to involve asynchronous vesicular release, recruits several different vesicular pools, leading to an overall recovery of 2 s. In addition, the depletion is so strong that it takes a total of 30 seconds to recover to the basal state. At this stage, we have shown that synaptic depression-facilitation accounts for the burst reverberation, which was a possibility left open in [21], while the authors rules out many intrinsic channel properties of the neurons. While synaptic depression is the dominant factor that prevented the fast recovery, it is also responsible for shutting down the burst activity as initially reported in [6].

### 0.2 The spontaneous network activity does not elicit a network burst comparable to an induced reverberation

Next, we extracted the spontaneous properties of the neuronal ensemble. For that purpose, we use the fluctuation dynamics of the bursting time. We thus add a source of noise in the first equation of system Eq 1

$$\begin{array}{c} \tau \dot{h}=-h+Jxyh^{+}+\tau H\delta \left(t-t_{stim}\right)+\sqrt{\tau }\sigma \dot{\omega }, \end{array}$$ (2)

where *ω* is a Gaussian white noise centered at zero of variance 1. By comparing the fluctuations of the burst duration obtained in the simulations and the experimental data, we extracted an amplitude of the neuronal noise of the order of *σ* = 2*Hz*. We further obtained (Fig. B (A) in S1 File) the distribution of the burst duration for the first and the second evoked pulses: the distribution of the first stimulation (centered at 2 s) is smaller compared to the second one (centered at 1s), suggesting that the first stimulation leads to a more robust response. We further found that the mean burst duration was not much sensitive to the network noise amplitude (Fig. A in S1 File). However, the duration of the first burst was slightly decreasing as a function of the noise, because the network was increasingly depressed (Fig. A in S1 File). Finally, we ran numerical simulations to study whether the spontaneous activity of the network (Fig. B in S1 File) could lead to a spontaneous event with an amplitude comparable to an induced burst. We conclude that with the amount of noise we extracted in the neuronal islands, the neuronal network cannot generate spontaneous bursting in time scale of 10 of minutes. Spontaneous depolarization generated by the noise is not sufficiently strong to induce a long time burst at a time scale of minutes, because the noise amplitude is too small. Generating a Burst is a rare event requiring a much longer time scale. This is the reason why we do not see them in the model, nor in the culture at this time scale. This analysis confirms that for such extracted noise amplitude, Burst can only be induced here by a local strong depolarization.

### 0.3 Estimating the reverberation time as a function of the network parameters

Because the burst duration depends strongly on the synaptic properties, we decided to investigate the effect of changing the total synaptic connections (variable J). For that purpose, because the model 1 could reproduce the bursting dynamics as observed experimentally, we decided to estimate analytically the reverberation time *T*_*R*_ by analyzing the equations. Thus, following the experimental protocol, an induced spike at time *t* = 0 sets the firing rate *h* to a value *H* and the reverberation time *T*_*R*_ is thus defined as the first time the firing rate *h* reaches a threshold value that we set equal to *h*_*T*_ = 10*Hz*, after which, we consider that there is no more any synchronous bursting activity. Thus, we define the reverberation time as

$$\begin{array}{c} T_{R}=\inf\left\{t>0,\;h\left(t\right)=h_{T}\;\text{when}\;\text{the}\;\text{stimulation}\;\text{is}\;\text{given}\;\text{at}\;\text{time}\;t=0\right\}. \end{array}$$ (3)

By definition, the burst occurs between 0 and *T*_*R*_. To estimate *T*_*R*_, we approximate during the early bursting period *t* ≪ *T*_*R*_ the firing rate *h*(*t*) ≈ *H* by a constant in the last two equations of system (Eq 1) (Figs. C and D in S1 File). Although this approximation affects the dynamics of the depression *x* and facilitation *y* variable, the decay phase of *h* is not much impacted. Within these approximations, we obtain (see Supplementary material) that the firing rate can be expressed as the depression and facilitation variables

$$\begin{array}{c} h\left(t\right)=H\exp(-\frac{t}{\tau }+J\int_{0}^{t}x\left(s\right)y\left(s\right)ds). \end{array}$$ (4)

The integral $\int_{0}^{t}x(s)y(s)ds$ can be estimated in terms of the variable $-t_{r}LHX(1+\frac{KH}{1/t_{f}+KH})$. By inverting equation *h*(*T*_*R*_) = *h*_*T*_ (the details are given for completeness in the S1 File), we obtain that new formula

$$\begin{array}{ccc} T_{R}\left(J\right) & = & \frac{\theta -\sqrt{\theta^{2}-2J\tau XH\left(K-LX\right)\tau \ln(\frac{H}{h_{th}})}}{J\tau XH(K-LX)}\\ \theta & = & 1-JX\tau . \end{array}$$ (5)

showing that the reverberation *T*_*R*_ is a sub-linear function of the synaptic connectivity J (Fig. E-F in S1 File). This formula synthesize the role of various parameters in shaping the bursting time. In particular, as J increases, the reverberation time starts increasing slowly, which was an unexpected property.

**Fig 3:**
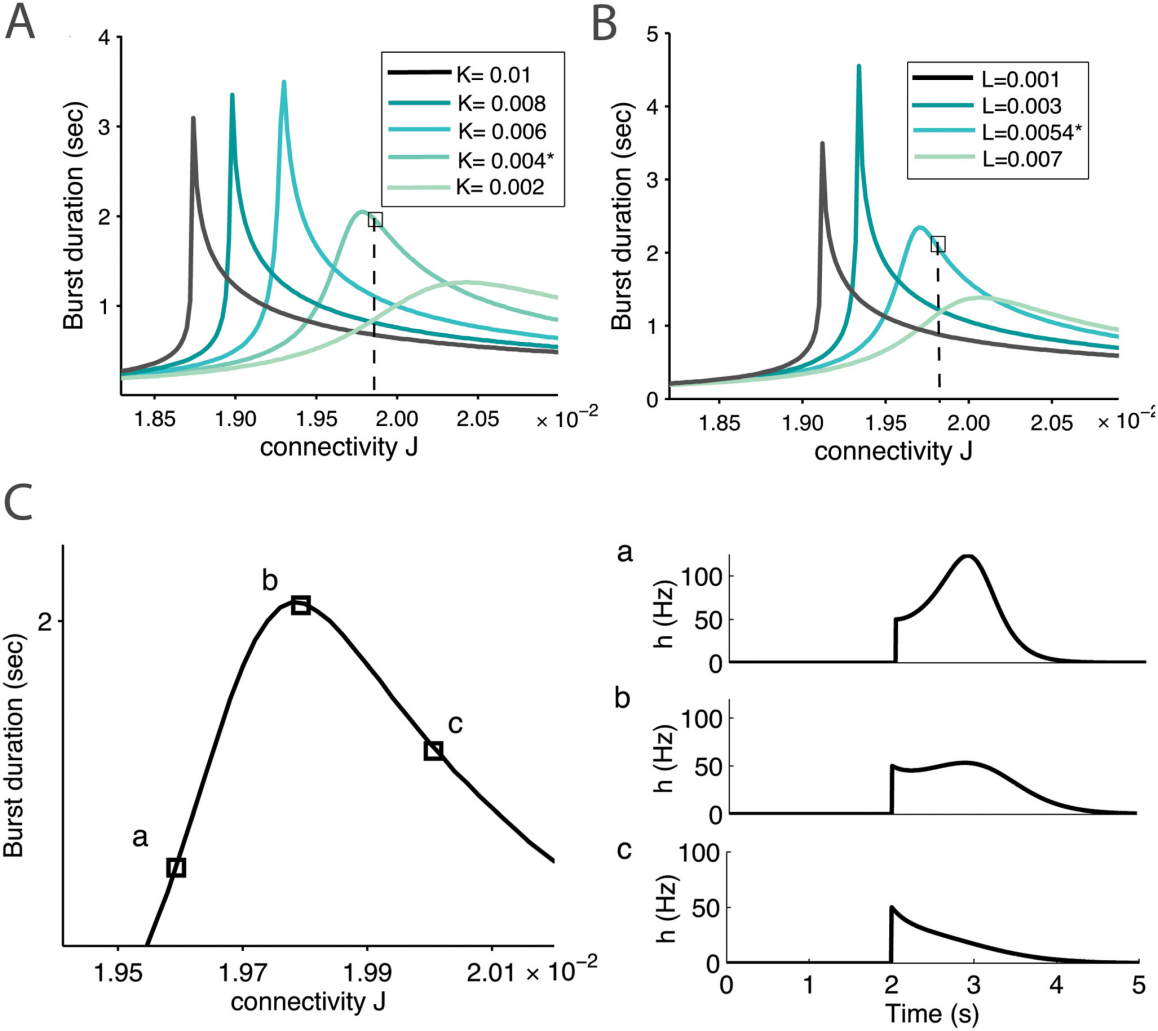
Reverberation time as a function of synaptic connectivity. *(A)* Burst duration time as function of the network connectivity (parameter *J*) for different values of the facilitation parameter *K* and *(B)* the depression parameter *L*, (other parameters are described in Table 1). We indicate the position of variable extracted for the experimental datas, which lie close to the maximum. *(C)* Burst shapes at three different stages of the bell shape curve (parameter L* and K*).

To investigate how the reverberation time *T*_*R*_ changes in a larger interval of synaptic connection, we use a numerical method and solve the complete system of Eq 1. We obtain a sublinear regime and interestingly, found that the slow increasing phase is followed by a quick decreasing phase, separated by a unique maximum value (Fig 3). Such a bell shape behavior can be interpreted as follows: when the network is not connected enough through synapses, facilitation cannot sustain a strong reverberation time, while in a network showing too many connections, synaptic depression dominates, preventing a long reverberation time. Thus we found an optimal reverberation time, occurring for a single value of the mean connectivity *J*.

Surprisingly, for the network parameters that accounted for the experimental data (Fig 2), we found that the parameters associated with the bursting time *T*_*R*_ are located close to the optimal value (Fig 3). It is not clear whether this is a pure coincidence or the optimum corresponds to an attractor in the development of the neuronal islands. We conclude that to obtain a maximal bursting time, the network should not be too much connected and this optimal reverberation time depends on the biophysical properties of the synapses and on the network connectivity.

### 0.4 The model could also account for decreasing extracellular calcium, that alters differentially and sequentially evoked bursts in small networks

**Fig 4:**
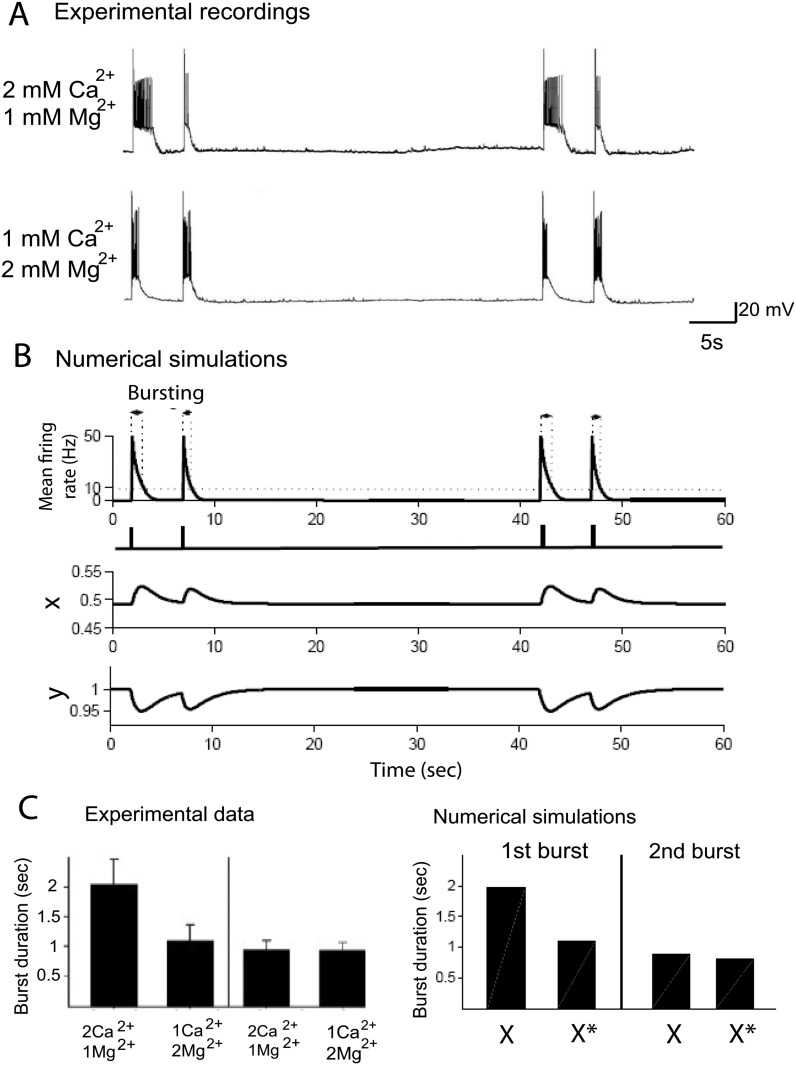
Calcium-dependence of reverberation bursts in small networks. *(A)* Evoked bursts recorded after exchanging the medium from 2 mM CaCl_2_ and 1 mM MgCl_2_ to 1 mM CaCl_2_ and 2 mM MgCl_2_. *(B)* Simulated response to evoked bursts: the extracellular concentration change is modeled by adjusting the parameter *X* to approximate the burst duration variation within the calcium concentration decrease. *(C)* Comparison of the burst durations demonstrating a reduction in the duration of the 1st burst (35 seconds interval burst) but not the 2nd burst (5 seconds interval burst), when [*Ca* ^2+^] was reduced and [*Mg* ^2+^] increased. We compare the first and second burst durations of the model for value of *X* = 0.50 given in Table 1 and X* = 0.4925, which account for the burst durations variations due to calcium concentration changes observed in left.

Changes in extracellular calcium levels (from 2 to 1 mM) in cultured hippocampal neurons led to a reduction of the mean duration time of the first burst, but not the second (Fig 4A and 4C and table B in S1 File). Thus to analyze the underlying mechanism, we used the present depression-facilitation model. Indeed, synaptic vesicular release is mediated in large by calcium-dependent mechanisms and is correlated with calcium transients in presynaptic terminals [25]. In our model, as synchronous EPSCs are generated, synaptic facilitation (variable x in system 1) increase reflects that calcium concentration is built up in the presynaptic terminal and as a result, it increases the vesicular release probability. Thus, in principle, changing the extracellular calcium concentration should affect the steady state facilitation steady state (parameter X in Eq 1), such that $X=X(Ca_{{}^{{}_{ex}}}^{0})+a(Ca_{ex}-Ca_{{}^{{}_{ex}}}^{0})$, where $Ca_{{}^{{}_{ex}}}^{0}$ is the initial extracellular calcium concentration and *Ca*_*ex*_ is the new one.

To account for the extracellular calcium decay, we diminished the parameter X (from 0.5 to a value 0.4925), which led to a reduction of the first mean duration time, while the second burst time was not affected, in agreement with the experimental data. The effect of changing the parameter *X* on the release probability becomes clear when comparing the dynamics of the facilitation variable *x*(*t*) (the probability is proportional to x(t)), during the bursts: in control conditions, the facilitation variable *x*(*t*) varies in the range [0.5–0.75] (Fig 2b), while after changing X to 0.4925, it varies in the range [0.49–0.53], this large difference explains that a small change in *X* affects drastically the second burst duration. The parameter *X* is singular: a small variation leads to a large change in the network dynamics. This effect is intrinsic to all depression-facilitation models. The interpretation of *X* is certainly link to calcium, where it is known that small fluctuations in the residual calcium affect the release probability. Thus in that context, the models reflects this specific non-robust property. New modelings and analysis at a biophysical level are certainly needed to better understanding how the parameter *X* is linked to biophysical quantities such as the residual calcium.

To conclude, this change on only one parameter allows to account for three constraints (histogram of the first, second (after 5s) and third burst(35s)), demonstrating the robustness of the model. Finally, the very small change in the variable *X* shows that it is certainly a singular parameter with respect to the steady calcium concentration, the dependency of which should be certainly studied in future works. In Fig 4B–4C, we show an agreement between the simulated and experimental burst duration. Indeed, we have shown here that the duration of the first evoked burst is predominantly controlled by synaptic facilitation, which is regulated by the extracellular calcium concentration.

### 0.5 Reverberation in larger scale neuronal network

**Fig 5:**
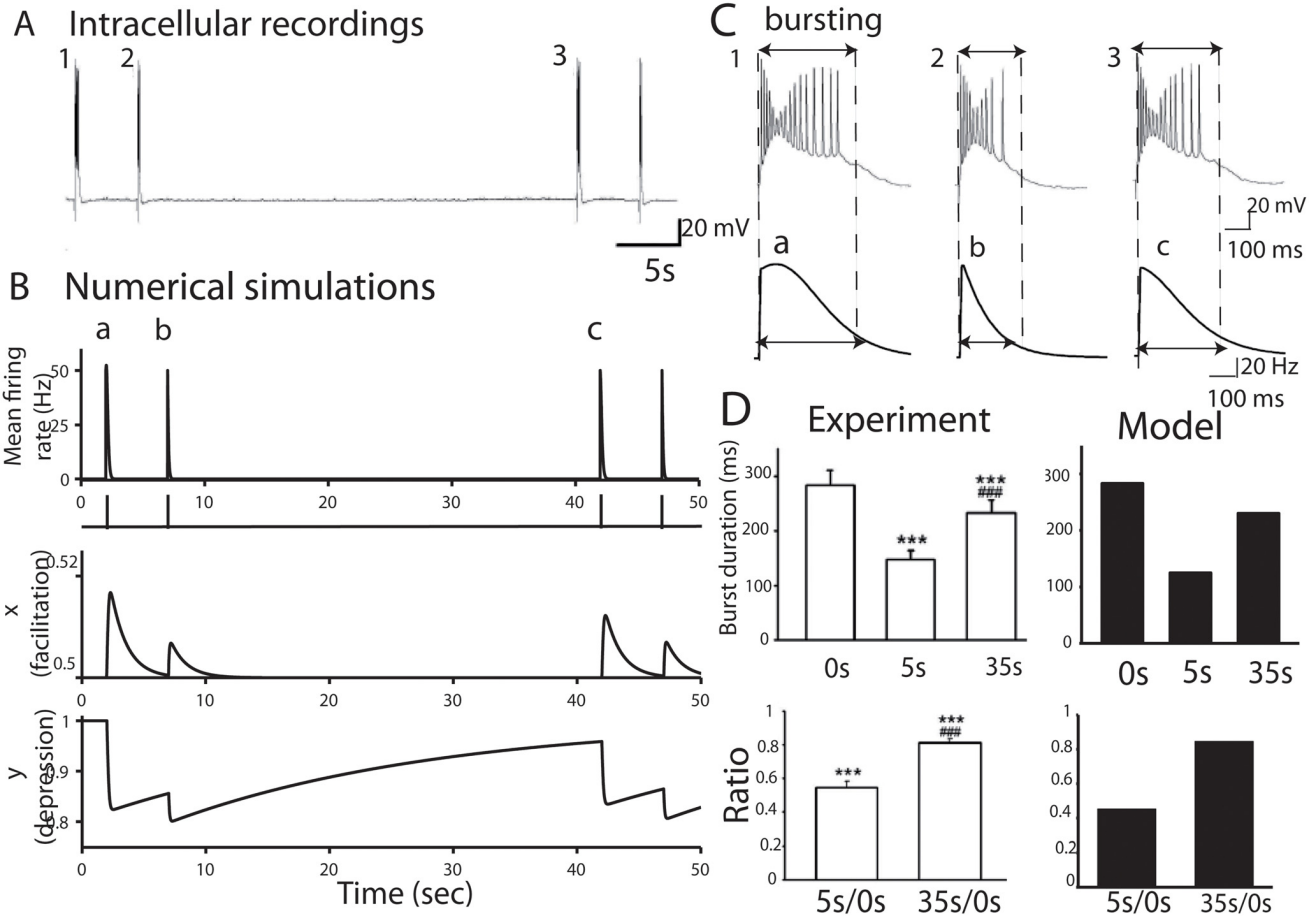
Reduction of burst duration in hippocampal slices and facilitation-depression synaptic model. *(A)* Bursts (1,2 and 3) in pyramidal cells from hippocampal acute slice evoked by a single stimulation of Schaffer collaterals alternately at 5 and 35 seconds intervals, showing a reduction in burst duration when the bursts are generated in shorter intervals. *(B)* Following the experimental protocol, we simulated the mean firing rate response (Eq 1), and generating bursts at similar time intervals (a, b and c) as in the intracellular recordings. The facilitation and depression variables show the level of neuronal activity underlying the overall dynamics. *(C)* Magnification of the evoked bursts (from A1, 2 and 3) and the simulated response (B a, b and c). *(D)* (Up) Comparison between experimental data and numerical simulations of bursting durations at the initial stimulation time, 5 and 35 s intervals. (Down) Burst duration ratios of the 2nd and 3rd to the initial burst (experimental data and numerical simulations).

To confirm that bursting reverberation was not only present in small neuronal islands, but could also be generated in integrated networks taken from brain tissue, we recorded pyramidal cells from acute hippocampal slices. By stimulating Schaffer collaterals, we were able to generate a burst that lasted 283.6 ± 26.9 ms (n = 22) (Fig 5A and table S1), which matched (Fig 5B–5C) the numerical simulations from the depression-facilitation model (Eq 1), where the new parameters are defined in Table 1. We compared in Fig 5D, the statistics of experimental data and numerical simulations of bursting durations at the initial stimulation times 5 and 35 s. The model reveals the higher degree of connectivity J and the longer depression time *t*_*r*_ = 20*s*.

### 0.6 The bursting duration depends on synaptic AMPA receptors

**Fig 6:**
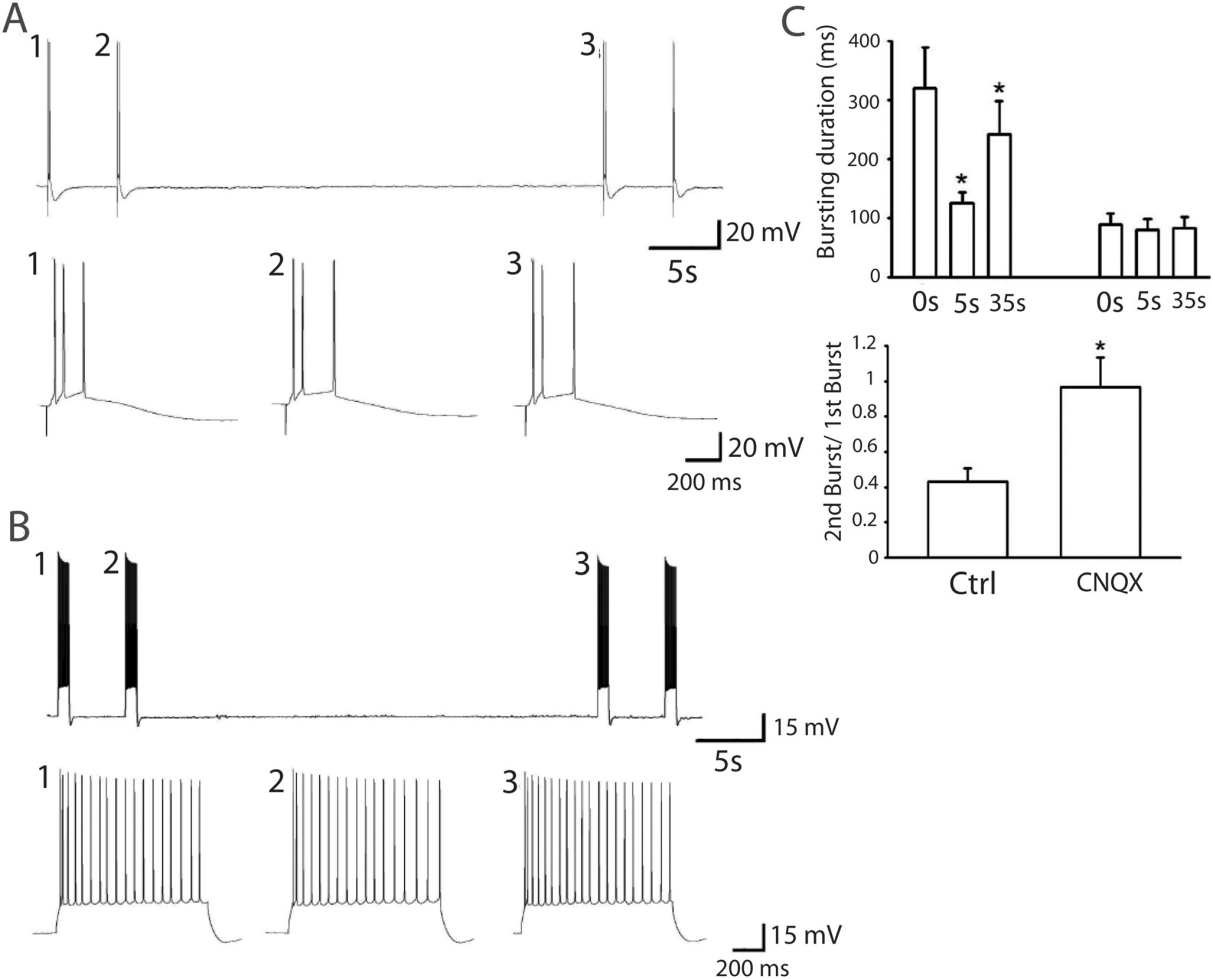
Calcium-dependence of reverberation bursts in large networks. *(A)* Evoked burst triggered by a single stimulation of Schaffer collaterals in hippocampal slices at 5 and 35 s intervals in the presence of low [Ca^2+^] solution (1.3 mM [Ca^2+^] and 2.5 mM [Mg^2+^]). *(B)* Comparison of the burst durations for two different calcium concentrations, leading to a reduction of the 1st burst duration (35 seconds interval burst) but not the 2nd burst (5 seconds interval burst), after low [Ca^2+^] solution application. (*P < 0.05, compared with 0 s, Student’s paired t-test). *(C)* Calcium reduction is modeled by changing the parameter *X*, which determines the steady state value of the facilitation variable *x*. *(D)* First and second burst durations for value of *X* = 0.50 (control Table 1) and *X* = 0.4925, which describes the burst duration variations due to calcium concentration changes observed in *A* and *B*.

To confirm the synaptic origin of the reverberation, we used CNQX (AMPA receptor antagonist, 1 *μ*M) that eliminated the bursting reverberation (Fig 6A and 6B). Thus the reverberation time involves AMPA receptors. Finally, to confirm the role of presynaptic neurons in generating the reverberation, we directly injected a current of 100 pA into the patched pyramidal neuron to trigger bursting, thus bypassing presynaptic activation. This did not induce depression in bursts evoked at 5 and 35 s intervals (Fig 6). Hence bursting duration requires both AMPA receptor dynamics and activation of presynaptic neurons.

### 0.7 Bursting reverberation is dependent on extracellular calcium levels in large networks

**Fig 7:**
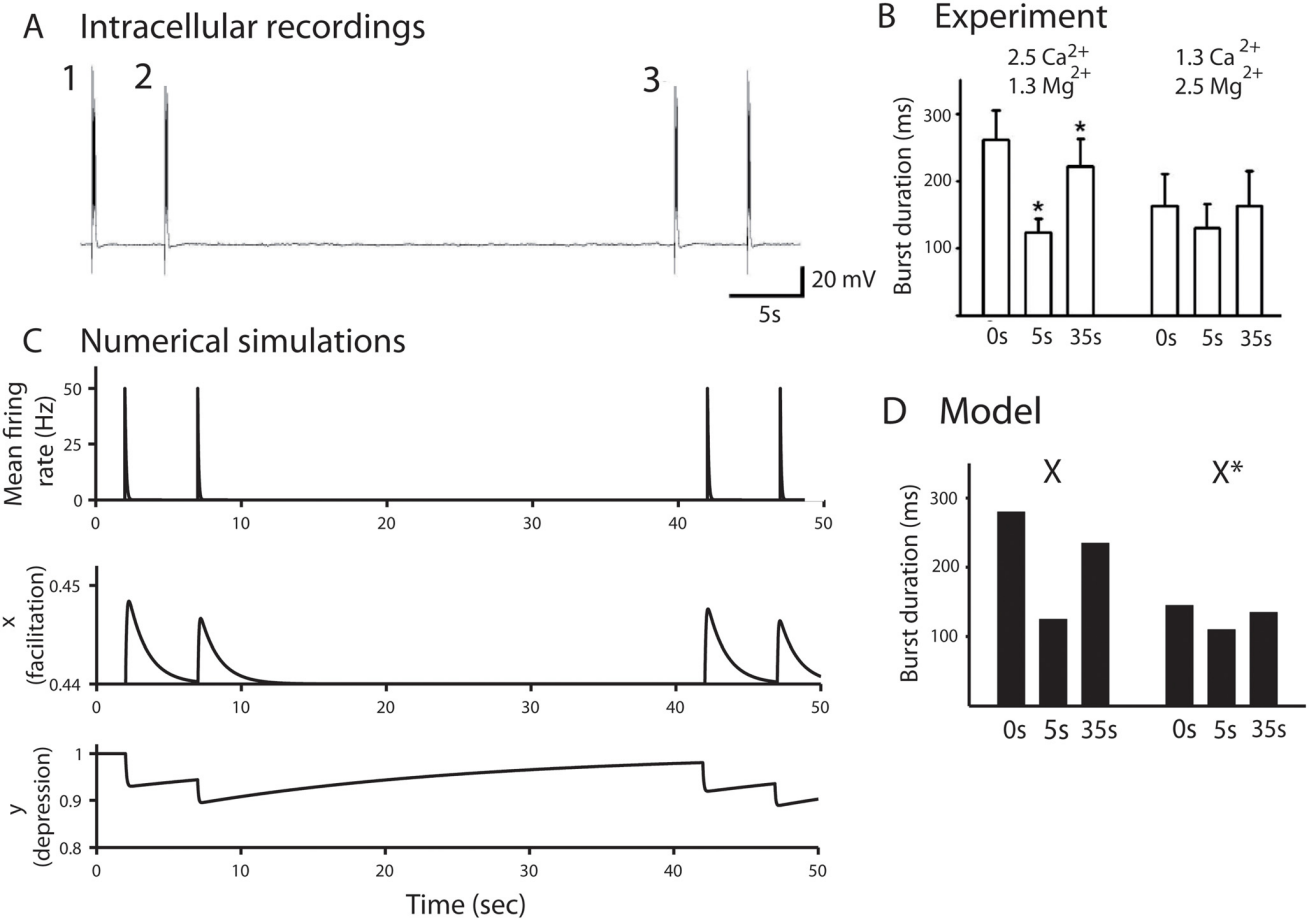
The bursting duration in slices depends on synaptic AMPA receptors. *(A)* CNQX (1 *μ*M) eliminated the bursting reverberation. *(B)* Bursting duration at 0, 5, and 35 s before and after CNQX application. (*P < 0.05, compared with 0 s, Student’s paired t-test). Ratio of bursting duration at 5 s before and after CNQX application (*P < 0.05, compared with control, Student’s paired t-test, n = 4). *(C)* Injection of 100 pA positive current into the patched pyramidal neuron triggered bursting without depression in 5 and 35 s interval, confirming that the bursting duration is synaptically dependent.

To further investigate the properties of the reverberation, we evoked a burst triggered by a single synaptic stimulation at 5 and 35 s intervals in the presence of low extracellular calcium (1.3 mM [Ca^2+^] and 2.5 mM [Mg^2+^]). We found a reduction in the duration of the first burst, but not the second one, as reported in the islands of neurons (Fig 7). A similar reduction is obtained in the depression-facilitation model when we change the associated parameter *X* (as discussed in section 4) which reflects the change in the steady state calcium concentration (Fig 7 and table B in S1 File), confirming that changes in burst reverberation due to a change in the extracellular calcium concentration affects facilitation.

### 0.8 Blocking energy metabolism of astrocytes does not affect bursting reverberation

Astrocytes have been proposed to contribute to postburst depression of release probability at CA3-CA1 excitatory synapses [26, 27]. Hence, we first investigated whether astrocytes respond to the evoked neuronal bursting. We found that astrocytes display depolarization of their membrane potential synchronously to neuronal bursting (Fig. C in S1 File). We then tested whether the burst long-lasting depression that we found in hippocampal slices was due to astrocytes, by evoking bursts before and after application of the astroglial metabolic poison FAC (fluoroacetate, 5 mM). However, neuronal bursting was unchanged by inhibition of astroglial metabolism. Hence, these data suggest that astrocytes are not directly involved in controlling bursting reverberation.

## Discussion

### Synaptic properties shape synchronous neuronal bursting and reverberation time

The physiological data obtained from small neural networks and the computational simulations reveal that the bursting reverberation essentially depends on synaptic properties. The contribution of the neuronal electrical property (involving channels, membrane capacity,.) in shaping the bursting time was already ruled out in [9]. From our modeling, we found here that synaptic facilitation at synapses is responsible for prolonging the network activation and it decays with a time scale of *τ*_*f*_ = 1.3 s, while with further stimulation, synaptic depression dominates the network recovery with a decay time of the order of *t*_*r*_ = 2 s (culture) and *t*_*r*_ = 20 s (slices). These parameters could vary with neuron types and for cortical neurons, the depression rate was estimated to be 10 times slower *τ*_*d*_ = 0.2 s [15], suggesting that various neurons are characterized by specific facilitation-depression parameters. Finally, the time course of the depression variable we have reported here is in agreement with fluorescence imaging and electrophysiology data [28, 29].

**Fig 8:**
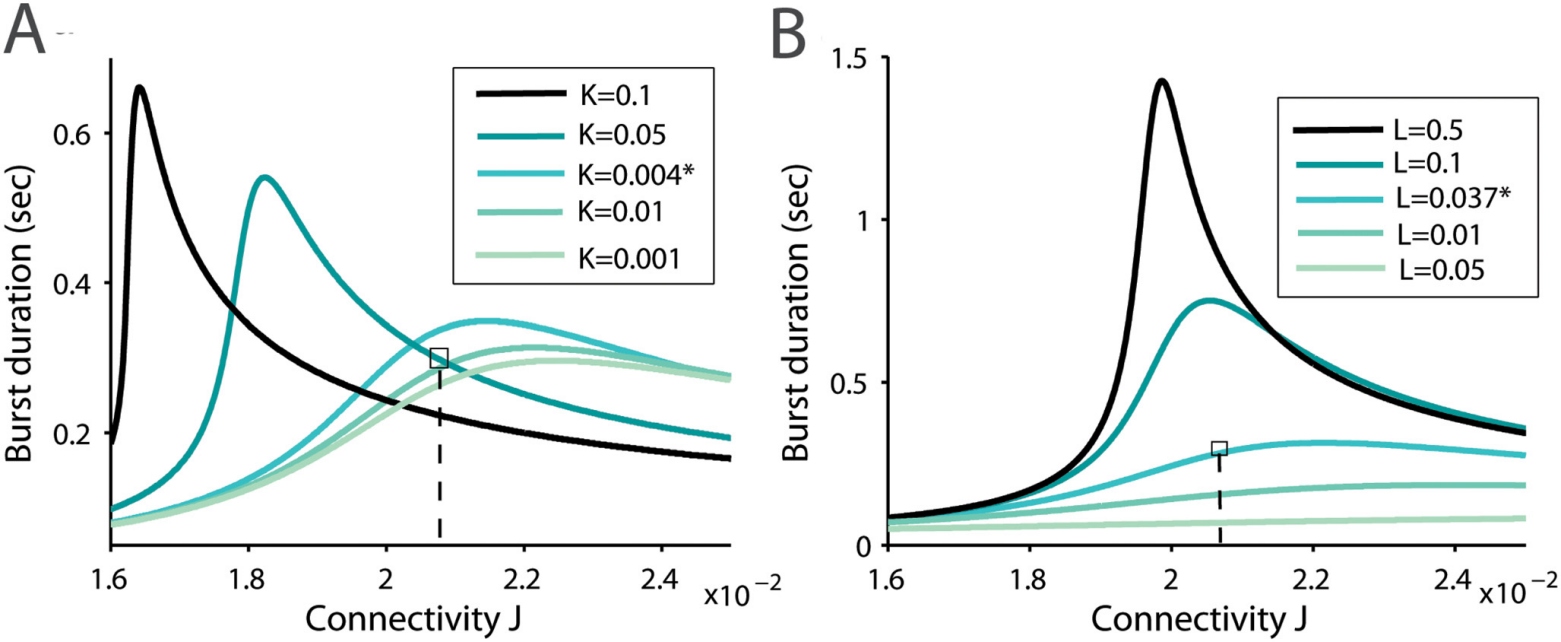
Reverberation time as a function of the synaptic connectivity (Slices). *(A)* Burst duration time as function of the network connectivity (parameter *J*) for different values of the facilitation parameter *K* and *(B)* the depression parameter *L*, (other parameters are described in Table 1). We indicate the position of variable extracted for the experimental datas, which lie close to the maximum.

**Fig 9:**
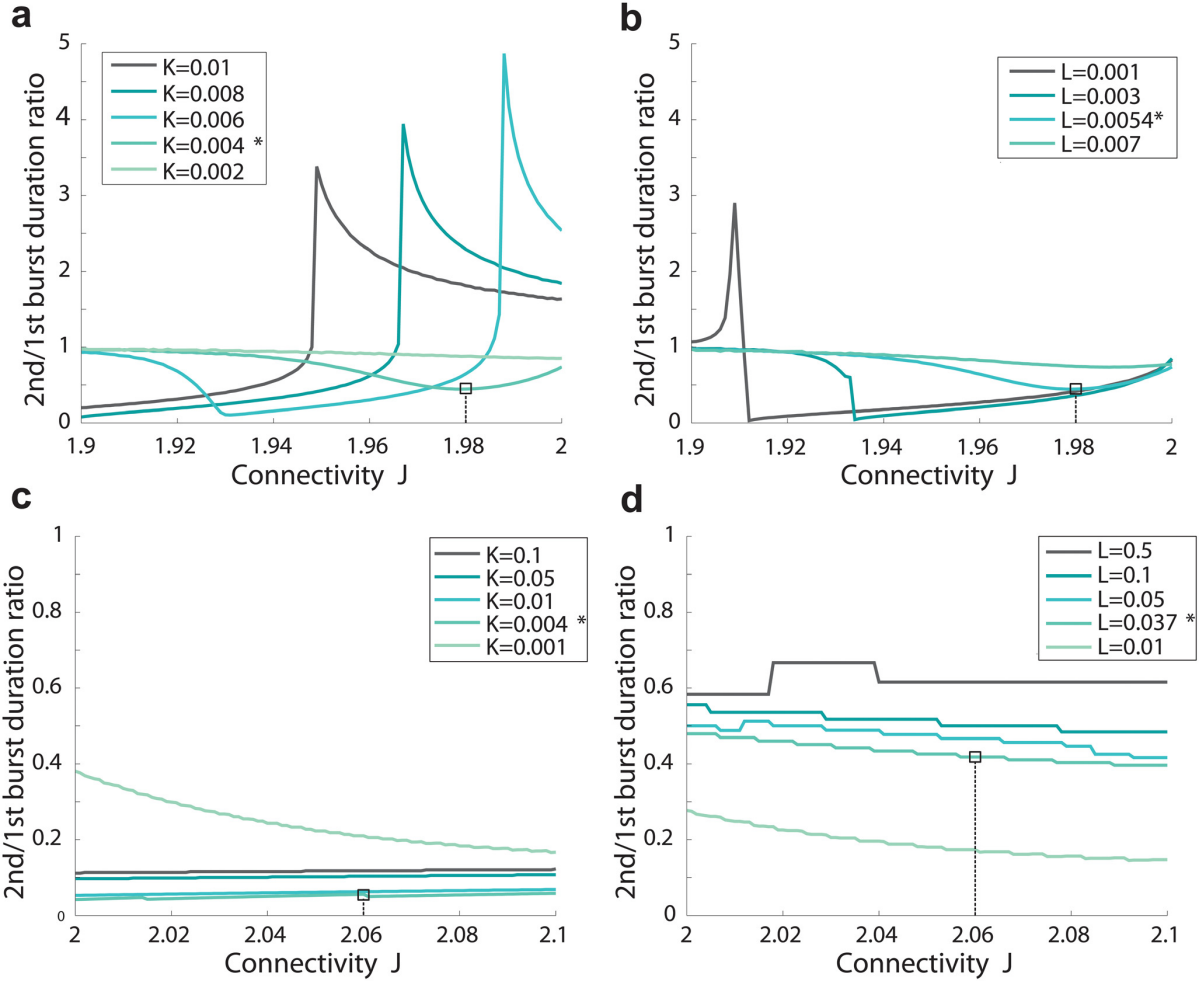
Ratio(2^*nd*^/1^*st*^) of Reverberation duration time as a function of the synaptic connectivity. (A) Culture (B) Slices.

From the depression-facilitation synaptic properties, we found that there is an optimal network connection, which sustains optimal burst duration (Fig 3). This prediction remains to be tested experimentally. We summarized in Fig 8 changes with neuronal connectivity of burst durations obtained for neural network in slices. The ratio of the second to the first burst with respect to the total synaptic connectivity is shown in Fig 9 and present clear differences of evolution between Culture and slices.

Furthermore, synaptic facilitation seems to be triggered by calcium dynamics, since changing the extracellular calcium concentration in both culture and slices (Figs 4 and 7) affected the first burst duration, but not the second one. Using our modeling approach, we conclude that this change in external calcium alters the steady state facilitation (X variable) in the second equation in 1. This internal state, which is calcium dependent, determines the length of the first burst duration, but does not influence the depression properties. This variable is very sensitive, suggesting that it depends on singular perturbation manner on the real physical parameters.

We confronted here the classical depression-facilitation model 1 [15] to experimental data recorded from network at various sizes, from which we estimated the associated parameters. In previous studies, this model was studied either theoretically [15] or applied to characterize synaptic properties of directly connected neurons. Using this framework, we have now found some parameters for depression, facilitation and network connectivity that can reproduce bursting for different size neural networks. We conclude that the depression-facilitation dynamics can describe bursting in neural networks of various sizes. It is possible that intrinsic properties interact with the network mechanism in bursting and depression, but the present study, as well as experimental evidence [21], indicate that depletion of synaptic vesicles is the primary reason for the time dependent suppression of network bursts.

### Bursting Reverberation is an intrinsic property of sufficiently connected neuronal networks

Because the mean-field system of equations (system 1) does not depend on the number of neurons, but rather on the density of synapses per neurons, we conclude that the bursting reverberation present in our model does not depend on the size of the neuronal ensemble, but rather on its degree of connectivity (parameter J), a prediction that we found in culture and slices. Indeed, we found that the bursting reverberation occurs also for large ensemble of connected neurons (slices). In addition, a similar model was recently used to analyze working memory behavior, which enables the temporary holding of information in the brain for several seconds. It was suggested that calcium mediates synaptic facilitation in the recurrent connections of neocortical networks [15], and here we confirm this property in small and larger neural networks. Furthermore, we use the invariance by scaling of our model to infer that reverberation should further persist in any larger networks, possibly underlying higher brain function.

### The average synaptic connectivity determines the bursting reverberation time

Using the present modeling, we have found that increasing synaptic strength *J* in Eq 1 (Fig 3 and C in S1 File), which reflects the number of functional synapses between neurons, is associated with increasing the reverberation time duration in a certain range, before the maximum is achieved. Synaptic plasticity is also associated with an increase in synaptic strength and in the mean number of connections. Our result suggests that although synaptic formation might increase linearly in a long-term learning process, the bursting window, which characterizes flash memory, is first small and the size increases sublinearly before it reaches a maximum, where a large number of synapses are required to observe a significant change in the bursting response time (Fig 3 and C in S1 file). This is certainly reminiscent of the nonlinear process associated with learning.

It is also intriguing that the islands of neuronal network that we have characterized operate in a regime close to the optimal burst response (Fig 3) and it would be interesting to determine what are the mechanisms that ensure the stability of the neural network at this value. This can either be due to internal neuronal dynamics or maintained by the network spontaneous activity. Furthermore, although the spontaneous activity did not contribute much in small neurons, it could have much more consequences in larger neuronal ensemble leading to spontaneous Up-states for example.

Finally, changing the depression and facilitation properties of synapses drastically affects the network connectivity, leading to a change in the amount of synapses associated with the maximal burst duration. We propose that pathological synapses, where the facilitation-depression properties are modified, will affect the neural network properties, especially associated with a significant change in bursting duration for small neuronal ensembles and with the optimal working memory response time for larger groups. It would be interesting to test whether such depression-facilitation properties are modified in some brain disorders and how it affects the burst duration.

## Materials and Methods

**Electrophysiology in hippocampal microcultures**. The methods are detailed elsewhere [9]. In brief, dissociated hippocampal neurons, diluted to 5–7 × 104 cells per ml, were plated on agarose-covered glass coverslips sprayed with fine droplets of substrate solution containing rat tail collagen (BD biosciences) at 1 mg/ml and poly-D-lysine (Sigma) at 0.3 mg/ml. These permissive islands allow the growth of networks of 5–30 neurons, which make extensive intrinsic connections.

At 2–3 weeks in culture, neurons were recorded in standard HEPES-buffered medium, containing 2 mM CaCl_2_ and 1mM MgCl_2_. Patch pipettes contained biocytin (0.4%) to visualize the neurons after termination of the experiments. In some experiments Alexa-Fluor 555 was added to the internal medium to visualize live neurons. Bursts were evoked either by injecting 5 ms depolarizing current pulses sufficient to evoke a single action potential in one neuron in current-clamp mode or by a 5 ms depolarization to 0 mV in voltage-clamp mode.

Network burst duration was estimated empirically as the time from the onset of the evoked action potential generating the burst to the time point when the falling phase of the polysynaptic current (PSC) crossed the threshold (at half of the averaged PSC amplitude). Signals were amplified with a Multiclamp-700B amplifier and recorded with Clampex 9.2 software (Axon Instruments, Union City, CA). Experiments were approved by the Weizmann Institutional Animal Care and Use Committee in accordance with EU directives Protocol Number:02740413-2

**Electrophysiology in acute brain slices** Experiments were carried out according to the guidelines of the European Community Council Directives of January 1st 2013 (2010/63/EU) and were approved by the local animal welfare committee (certificate A751901, Ministere de l’Agriculture et de la Peche). All efforts were made to minimize the number of animals used and their suffering. Experiments were performed on 17- to 25-day old C57BL6 mice. For all analysis, mice of both genders and littermates were used. Acute transverse hippocampal slices (400 μm) were prepared as previously described ([30]). Slices were maintained at room temperature in a storage chamber perfused with an artificial cerebrospinal fluid (ACSF) (containing 119 mM NaCl, 2.5 mM KCl, 2.5 mM CaCl_2_, 1.3 mM MgSO_4_, 1 mM NaH_2_PO_4_, 26.2 mM NaHCO_3_, and 11 mM glucose, saturated with 95% O_2_ and 5% CO_2_, pH 7.4, 320–330 mOsm) for at least 1 h. Slices were transferred to another storage chamber with the same ACSF with additional 100 *μ*M picrotoxin at least 30 min before recording. Slices were then transferred to a submerged recording chamber mounted on an Olympus BX51WI microscope equipped for infrared-differential interference (IR-DIC) microscopy and were perfused with ACSF at a rate of 1.5–2 ml/min at room temperature. All experiments were performed in the presence of picrotoxin (100 *μ* M). Somatic whole-cell recordings were obtained from visually identified hippocampal CA3 and CA1 pyramidal cells, using 5–8 MΩ glass pipettes filled with 105 mM Kgluconate, 30 mM KCl, 10 mM HEPES, 10 mM phosphocreatine, 4 mM Mg_2_-ATP, 0.3 mM Tris-GTP, and 0.3 mM EGTA (pH 7.4, 280 mOsm). Synaptically-evoked action potential bursts were triggered by a stimulation of Schaffer collaterals (10 mA, 50 *μ*s) with an ACSF-filled glass pipette. Bursts were recorded in the presence of 100 *μ*M picrotoxin at a resting membrane potential of -70 ± 2 mV. The whole-cell recording pipette was placed 50–100 *μ*m away from the stimulation pipette. In the experiment of low [Ca^2+^]o, to keep extracellular divalent ion concentration constant, CaCl_2_ was reduced to 1.3 mM while MgSO_4_ was increased to 2.5 mM. Recordings were acquired with Multiclamp-700B amplifiers, digitized at 10 kHz, filtered at 2 kHz, and stored and analyzed on a computer using pClamp 10 and Clampfit 10 software. All data are expressed as mean ± SEM. Statistical significance for comparisons was determined by Student’s paired t-test. Picrotoxin was obtained from Sigma and CNQX was from Tocris.

All simulations were run with Matlab. The computation of the reverberation time is presented in the S1 File.

## Supporting Information

S1 File The Supporting Information contains:
Table A: Burst durations in island cultures and acute slices.Table B: Comparison of burst durations for different extracellular calcium concentrationsA derivation section for formula [5]: Analytical estimation of the reverberation time *T*_*R*_
5 figures described below:
**Figures in the Supporting Information file**
Figure A. Reverberation bursting ratio when the interval between pulses varies. Using the parameters for culture (see table A in S1 File, the ratio converges to one after ten seconds.Figure B. **Effect of noise on the reverberation burst.** *(A)* Burst duration after the first and the second pulse as a function of the noise amplitude *σ*, for each value of the noise amplitude *σ* (500 runs). *(B)* Numerical simulations of the evoked bursts, generated at 5 and 35 seconds intervals with a source noise, extracted from the experimental data (*σ* = 2 Hz). Spontaneous activity is not enough to generate a response comparable to the evoked one.Figure C. ** Blocking astroglial metabolism does not affect the bursting reverberation.** *(A)* Evoked burst triggered by a single synaptic stimulation with a 5 s interval in the presence of fluoroacetate (FAC, 5 mM). *(B)* Simultaneous depolarization of astrocyte during the bursting pulse. *(C)* Bursting duration at 0 and 5 s before and after FAC application. (**P < 0.01, compared with 0 s, Student’s paired t-test). *(D)* Ratio of bursting duration at 5 s before and after FAC application (*P* > 0.05, compared with control, Student’s paired t-test, n = 4).Figure D. **Comparison of system of Eq 1 (continuous line) and the approximated system 2 (dashed line)**. We use three different values of the connectivity parameter *J*. The firing rate *h*, the facilitation *x* and the depression *y* variables are plotted as functions of time. For a low enough connectivity parameter *J*, the firing rate is well approximated.Figure E. **Comparison between the depression variable *y* estimated by equation 8 (blue) and the exact one obtained by numerical simulation of system 2 (black).**
Figure F. **Comparison between numerical simulations and estimates of the reverberation time *T*_*R*_.** The reverberation time is plotted as a function of *J* for the exact model (solid line), the approximated model (dash black line), and the estimates given by equations 37 (dash red line) and 39 (blue dash line).
(PDF)

## References

[1] Fuster JM, Alexander GE (1971) Neuron activity related to short-term memory. *Science* 173: 652654 10.1126/science.173.3997.652 4998337

[2] Traub R, Miles R. Neuronal Networks of the Hippocampus, Cambridge 1991.

[3] Lau PM, Bi GQ (2005) Synaptic mechanisms of persistent reverberatory activity in neuronal networks. *Proc Natl Acad Sci USA*, 102, 29, 10333–10338 10.1073/pnas.0500717102 16006530 PMC1177363

[4] Wyart C, Cocco S, Bourdieu L, Leger JF, Herr C, Chatenay D (2005) Dynamics of excitatory synaptic components in sustained firing at low rates. *J Neurophysiol*, 93(6):3370–80. 10.1152/jn.00530.2004 15673554

[5] Sah P (1996) Ca(2+)-activated K+ currents in neurones: types, physiological roles and modulation. *Trends Neurosci* 19:150154. 10.1016/s0166-2236(96)80026-9 8658599

[6] Staley KJ, Longacher M, Bains JS, Yee A, Presynaptic modulation of CA3 network activity. Nat Neurosci. (1998);1(3):201–9. 10.1038/651 10195144

[7] Mills J, Pitman RM (1999) Contribution of potassium conductances to a time-dependent transition in electrical properties of a cockroach motoneuron soma. *J Neurophysiol* 81(5): 2253–2266 10322064 10.1152/jn.1999.81.5.2253

[8] Bacci A, Verderio C, Pravettoni E, Matteoli M (1999) Astrocytes are required for the oscillatory activity in cultured hippocampal neurons. *Eur J Neurosci* 11:389–397. 10.1046/j.1460-9568.1999.00440.x 10457176

[9] Cohen D, Segal M, (2009) Homeostatic presynaptic suppression of neuronal network bursts. *J. Neurophysiol* 101:2077–2088. 10.1152/jn.91085.2008 19193770

[10] Picardo MA, Guigue P, Bonifazi P, Batista-Brito R, Allene C, Ribas A, Fishell G, Baude A, Cossart R (2011) Pioneer GABA cells comprise a subpopulation of hub neurons in the developing hippocampus. *Neuron* 71(4):695–709. 10.1016/j.neuron.2011.06.018 21867885 PMC3163067

[11] Tsodyks MV, Markram H. Proc Natl Acad Sci U S A. 1997;94(2):719–23. Erratum in: Proc Natl Acad Sci U S A 1997;94(10):5495. 10.1073/pnas.94.2.719 9012851 PMC19580

[12] Wang XJ (2001) Synaptic reverberation underlying mnemonic persistent activity. *Trends Neurosci* 24(8):455–63. 10.1016/S0166-2236(00)01868-3 11476885

[13] Holcman D. and Tsodyks M (2006), The emergence of up and down states in cortical networks. *PLOS Comp. Biology*, 2(3):e23 10.1371/journal.pcbi.0020023 PMC1409813 16557293

[14] Bart E, Bao S, Holcman D. Modeling the spontaneous activity of the auditory cortex, J Comput Neurosci. 2005;19(3):357–78. 16502241 10.1007/s10827-005-3099-4

[15] Tsodyks M, Pawelzik K, Markram H (1998) Neural networks with dynamic synapses. *Neural Comput*, 10(4):821–35. 10.1162/089976698300017502 9573407

[16] Bressloff PC, Lai YM, (2011), Stochastic synchronization of neuronal populations with intrinsic and extrinsic noise. *J Math Neurosci*;1(1):2 10.1186/2190-8567-1-2 22656265 PMC3280892

[17] Brunel N Hakim V. (2008) Sparsely synchronized neuronal oscillations. Chaos: An Interdisciplinary Journal of Nonlinear Science 18:1, 015113 10.1063/1.2779858 18377094

[18] Katz B, Miledi R (1968) The role of calcium in neuromuscular facilitation. *J Physiol* 195 (2): 481492 10.1113/jphysiol.1968.sp008469 PMC1351674 4296699

[19] Muller M, Goutman J, Kochubey O, Schneggenburger R (2010) Interaction between facilitation and depression at a large CNS synapse reveals mechanisms of short-term plasticity. *J Neurosci* 30(6):20072016 10.1523/JNEUROSCI.4378-09.2010 PMC6634054 20147529

[20] Tabak J, Rinzel J, O’Donovan J (2001) Modeling of spontaneous activity in developing spinal cord using activity-dependent depression in an excitatory network. *J Neurosci* 21(22):8966–8978. 11698607 10.1523/JNEUROSCI.21-22-08966.2001 PMC6762295

[21] Cohen D, Segal M (2011) Network bursts in hippocampal microcultures are terminated by exhaustion of vesicle pools. *J Neurophysiol* 106(5):2314–21 10.1152/jn.00969.2010 21832037

[22] Melamed O Omi BG. Silberberg H. Markram M. Tsodyks, Slow oscillations in neural networks with facilitating synapses, J Comput Neurosci (2008) 25:308316 10.1007/s10827-008-0080-z 18483841

[23] Xu-Friedman MA, Regehr WG (2004) Structural contributions to short-term synaptic plasticity. *Physiol Rev*. 84(1):69–85. 10.1152/physrev.00016.2003 14715911

[24] Atluri P and Wade G. Regeh, The Journal of Neuroscience, 1996, 16 (18):5661–5671. 8795622 10.1523/JNEUROSCI.16-18-05661.1996 PMC6578977

[25] Xu J, Pang ZP, Shin OH, Sudhof TC (2009) Synaptotagmin-1 functions as a Ca2+ sensor for spontaneous release. *Nat. Neurosci*. 12:759–766. 10.1038/nn.2320 19412166 PMC2739891

[26] Andersson M, Hanse E (2010) Astrocytes impose postburst depression of release probability at hippocampal glutamate synapses. *J Neurosci* 21;30(16):5776–80. 10.1523/JNEUROSCI.3957-09.2010 20410129 PMC6632337

[27] Andersson MS, Hanse E (2011), Astrocyte-mediated short-term synaptic depression in the rat hippocampal CA1 area: two modes of decreasing release probability. *BMC Neurosci* 24;12:87 10.1186/1471-2202-12-87 21864406 PMC3178542

[28] Maeda H, Ellis-Davies GC, Ito K, Miyashita Y, Kasai H, (1999) Supra-linear Ca2+ signaling by cooperative and mobile Ca2+ buffering in Purkinje neurons. *Neuron* 4:989–1002. 10.1016/S0896-6273(00)81045-4 10624961

[29] Blatow M, Caputi A, Burnashev N, Monyer H, Rozov A, (2003) Ca2+ buffer saturation underlies paired pulse facilitation in calbindin-D28k-containing terminals. *Neuron* 38(1):79–88. 10.1016/S0896-6273(03)00196-X 12691666

[30] Pannasch U, Sibille J, Rouach N (2012) Dual electrophysiological recordings of synaptically-evoked astroglial and neuronal responses in acute hippocampal slices. *J Vis Exp* (69):e4418 10.3791/4418 23222635 PMC3564483

