# Correction: Bursting Reverberation as a Multiscale Neuronal Network Process Driven by Synaptic Depression-Facilitation

**DOI:** 10.1371/journal.pone.0137884

**Published:** 2015-09-03

**Authors:** The PLOS ONE Staff

The first and second authors’ names appear incorrectly in the author byline. The correct names are: Khanh Dao Duc and Chun-Yao Lee. The publisher apologizes for these errors. The correct citation is: Dao Duc K, Lee CY, Parutto P, Cohen D, Segal M, Rouach N, et al. (2015) Bursting Reverberation as a Multiscale Neuronal Network Process Driven by Synaptic Depression-Facilitation. PLoS ONE 10(5): e0124694. doi:10.1371/journal.pone.0124694

There are errors in Fig 6, “The bursting duration in slices depends on synaptic AMPA receptors.” Panel B should be labeled panel C, and panel C should be labeled panel B.

In addition, the captions for Figs 6 and 7 are incorrectly switched. The figure images appear in the correct order. Please see the corrected Figs 6 and 7 and their captions below.

**Fig 6:**
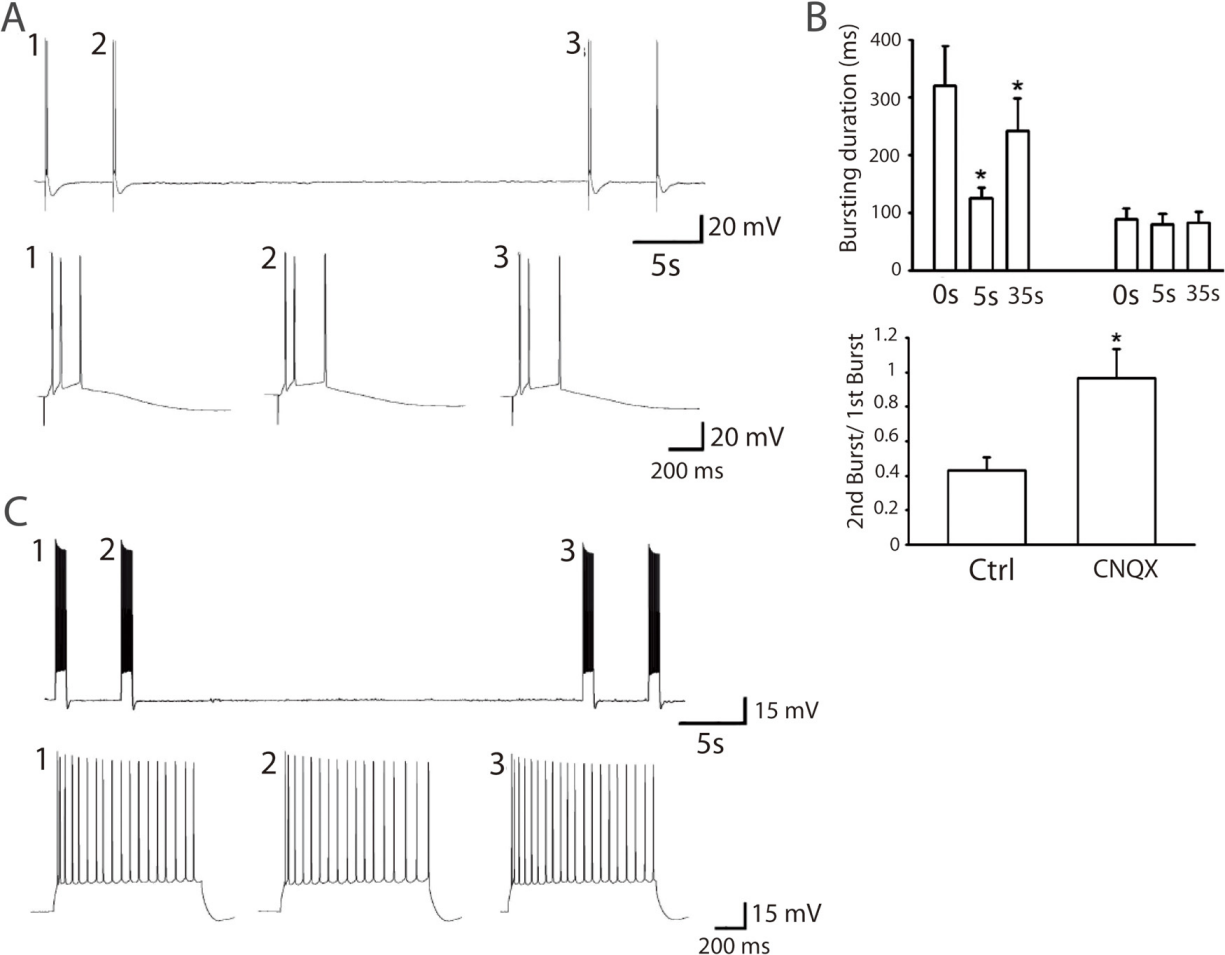
The bursting duration in slices depends on synaptic AMPA receptors. *(A)* CNQX (1 *μ*M) eliminated the bursting reverberation. *(B)* Bursting duration at 0, 5, and 35 s before and after CNQX application. (*P < 0.05, compared with 0 s, Student’s paired t-test). Ratio of bursting duration at 5 s before and after CNQX application (*P < 0.05, compared with control, Student’s paired t-test, n = 4). *(C)* Injection of 100 pA positive current into the patched pyramidal neuron triggered bursting without depression in 5 and 35 s interval, confirming that the bursting duration is synaptically dependent.

**Fig 7:**
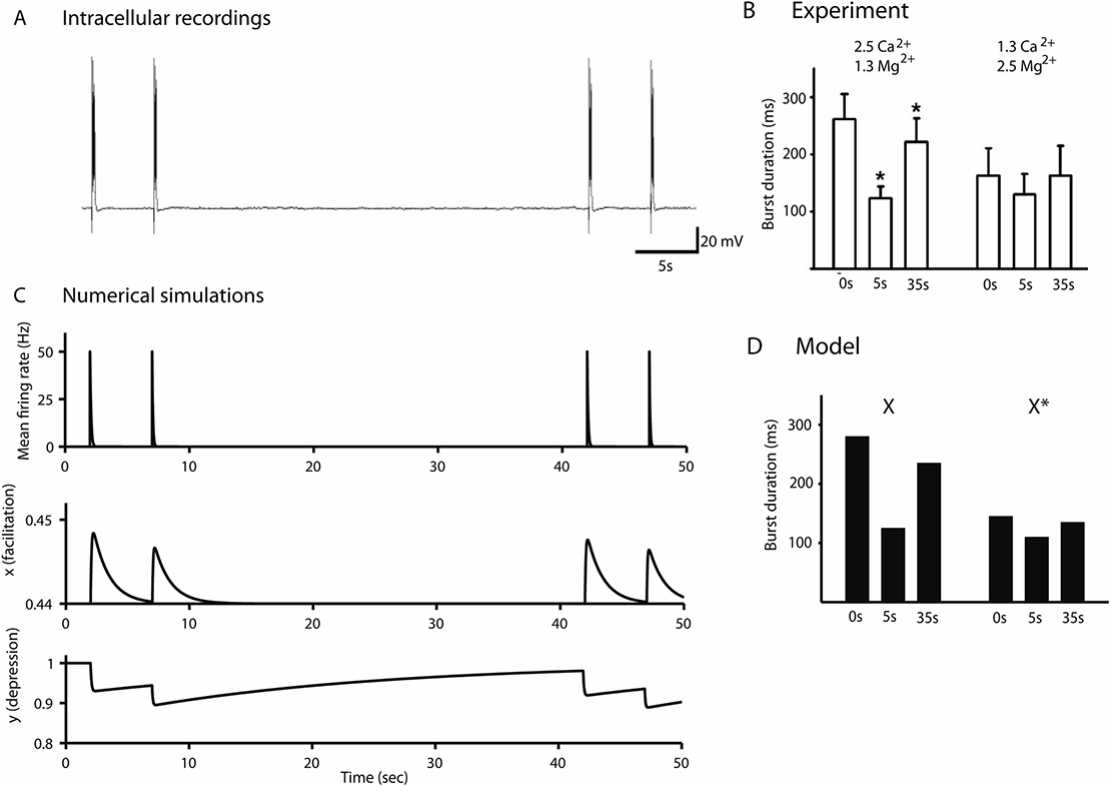
Calcium-dependence of reverberation bursts in large networks. *(A)* Evoked burst triggered by a single stimulation of Schaffer collaterals in hippocampal slices at 5 and 35 s intervals in the presence of low [Ca^2+^] solution (1.3 mM [Ca^2+^] and 2.5 mM [Mg^2+^]). *(B)* Comparison of the burst durations for two different calcium concentrations, leading to a reduction of the 1st burst duration (35 seconds interval burst) but not the 2nd burst (5 seconds interval burst), after low [Ca^2+^] solution application. (*P < 0.05, compared with 0 s, Student’s paired t-test). *(C)* Calcium reduction is modeled by changing the parameter *X*, which determines the steady state value of the facilitation variable *x*. *(D)* First and second burst durations for value of *X* = 0.50 (control Table 1) and *X* = 0.4925, which describes the burst duration variations due to calcium concentration changes observed in *A* and *B*.
