# Supplementary material for: Bursting Reverberation as a Multiscale Neuronal Network Process Driven by Synaptic Depression-Facilitation
Source: PLoS One. 2015 May 27;10(5):e0124694. doi: 10.1371/journal.pone.0124694 (PMC4446271; doi:10.1371/journal.pone.0124694)
Supplement: S1 File — The Supporting Information contains: Table A: Burst durations in island cultures and acute slices.Table B: Comparison of burst durations for different extracellular calcium concentrationsA derivation section for formula [5]: Analytical estimation of the reverberation time T R 5 figures described below: Figures in the Supporting Information file Figure A. Reverberation bursting ratio when the interval between pulses varies. Using the parameters for culture (see table A in S1 File, the ratio converges to one after ten seconds.Figure B. Effect of noise on the reverberation burst. (A) Burst duration after the first and the second pulse as a function of the noise amplitude σ, for each value of the noise amplitude σ (500 runs). (B) Numerical simulations of the evoked bursts, generated at 5 and 35 seconds intervals with a source noise, extracted from the experimental data (σ = 2 Hz). Spontaneous activity is not enough to generate a response comparable to the evoked one.Figure C. Blocking astroglial metabolism does not affect the bursting reverberation. (A) Evoked burst triggered by a single synaptic stimulation with a 5 s interval in the presence of fluoroacetate (FAC, 5 mM). (B) Simultaneous depolarization of astrocyte during the bursting pulse. (C) Bursting duration at 0 and 5 s before and after FAC application. (**P < 0.01, compared with 0 s, Student’s paired t-test). (D) Ratio of bursting duration at 5 s before and after FAC application (P > 0.05, compared with control, Student’s paired t-test, n = 4).Figure D. Comparison of system of Eq 1 (continuous line) and the approximated system 2 (dashed line). We use three different values of the connectivity parameter J. The firing rate h, the facilitation x and the depression y variables are plotted as functions of time. For a low enough connectivity parameter J, the firing rate is well approximated.Figure E. Comparison between the depression variable y estimated by equation 8 (blue) and the exact one obtained by numerical si [file pone.0124694.s001.pdf]

Supporting Information
Bursting reverberation as a multiscale neuronal network process, driven by synaptic depression-facilitation

K. Dao Duc, C.Y. Lee, D. Cohen,P. Parutto,
M. Segal, N. Rouach, D. Holcman

March 27, 2015

1 Introduction

In this supplementary material, we first present the tables of parameters used in the model for neuronal island and hippocampal slices. We then show that the level of the noise is not enough to generate spontaneous bursting at a time scale of minutes. Finally, we show that blocking the metabolism of astrocyte does not affect bursting reverberation. Finally, we show the analytical computation of the reverberation time as a function of the synaptic connectivity J.

Tables of Results

Table A: Burst durations in island cultures and acute slices.

Stimulation	Burst duration	Ratio with the first burst duration	Simulated burst duration	Ratio with the first burst duration
island cultures (n=20)				
5 s	0.99 ± 0.77 s[1]	0.49	0.92 s	0.45
35 s	2.26 ± 0.75 s[1]	1	2.045 s	1
acute slices (n=22)				
0 s	283.6 ± 26.9 ms	1	280 ms	1
5 s	147.8 ± 15.9 ms	0.55	125 ms	0.45
35 s	232.2 ± 24.3 ms	0.81	240 ms	0.85

Table B: Comparison of burst durations for different extracellular calcium concentrations

Interpulse interval (s)/ Calcium concentration(experiment)/ facilitation steady state (model)	Experimental burst duration	Simulated burst duration
island cultures (n=5)		
5 / 2 Ca^{2+}/X	0.98 ± 0.38 s [1]	0.92 s
35/ 2 Ca^{2+}/X	2.03 ± 0.98 s [1]	2.045 s
5 / 1 Ca^{2+}/X*	0.93 ± 0.32 s [1]	0.81 s
35/ 1 Ca^{2+}/X*	1.10 ± 0.62 s [1]	1.115 s
acute slices(n=5)		
0 / 2.5 Ca^{2+}/X	260.7 ± 44.2 ms	280 ms
5 / 2.5 Ca^{2+}/X	123.1 ± 20.7 ms	125 ms
35/ 2.5 Ca^{2+}/X	221.0 ± 41.5 ms	240 ms
0 / 1.3 Ca^{2+}/X*	162.6 ± 47.6 ms	165 ms
5 / 1.3 Ca^{2+}/X*	129.5 ± 36.4 ms	115 ms
35/ 1.3 Ca^{2+}/X*	162.1 ± 53.3 ms	145 ms

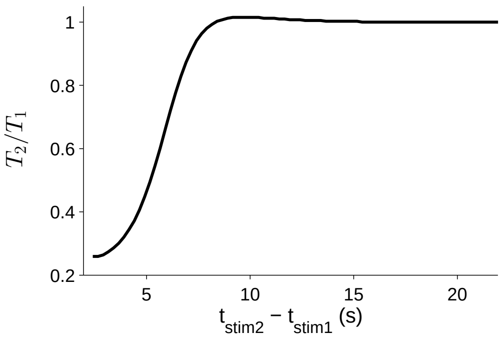

Figure A. Reverberation bursting ratio when the interval between pulses varies. Using the parameters for culture (see table 1, the ratio converges to one after ten seconds.

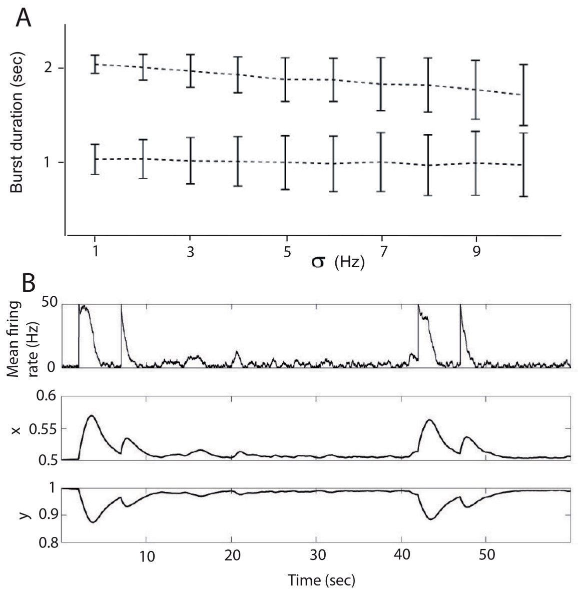

Figure B. Effect of noise on the reverberation burst. *(A)* Burst duration after the first and the second pulse as a function of the noise amplitude σ, for each value of the noise amplitude σ (500 runs). *(B)* Numerical simulations of the evoked bursts, generated at 5 and 35 seconds intervals with a source noise, extracted from the experimental data ($\sigma = 2$ Hz). Spontaneous activity is not enough to generate a response comparable to the evoked one.

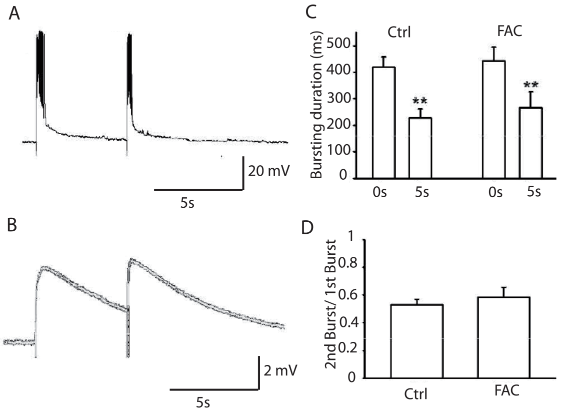

Figure C. Blocking astroglial metabolism does not affect the bursting reverberation. *(A)* Evoked burst triggered by a single synaptic stimulation with a 5 s interval in the presence of fluoroacetate (FAC, 5 mM). *(B)* Simultaneous depolarization of astrocyte during the bursting pulse. *(C)* Bursting duration at 0 and 5 s before and after FAC application. ($**P < 0.01$, compared with 0 s, Student's paired t-test). *(D)* Ratio of bursting duration at 5 s before and after FAC application ($P > 0.05$, compared with control, Student's paired t-test, n=4).

Derivation of formula [5]: Analytical estimation of the reverberation time T_R

We present in this section our analytical computation of the reverberation time as a function of the synaptic connectivity J (formula 5 of the main manuscript). This reverberation time T_R is defined using the firing rate variable h, as the duration of the bursting activity above a certain threshold h_{th}, induced here by a single spike. During the reverberation period, the firing rate remains approximatively constant in the initial phase of the response, which allows us to partially decouple the synaptic equations (system 1 in the

main text), that we recall now

$$\begin{aligned}
\tau\dot{h} &= -h + Jxyh + H\delta(t - t_{stim}) \\
\dot{x} &= \frac{X - x}{t_f} + K(1 - x)h \\
\dot{y} &= \frac{1 - y}{t_r} - Lxyh.
\end{aligned} \tag{1}$$

Our goal is to estimate T_R as a function of the threshold h_{th}.

Approximation procedure

During the early bursting time, we approximate equation 2 and 3 of system (1), by considering $h(t) \approx H$, its initial value and obtain the new approximated system

$$\begin{aligned}
\tau\dot{h} &= -h + Jxyh + \tau H\delta(t - t_{stim}) \\
\dot{x} &= \frac{X - x}{t_f} + K(1 - x)H \\
\dot{y} &= \frac{1 - y}{t_r} - LxyH.
\end{aligned} \tag{2}$$

Indeed, the firing rate h depends on the facilitation and depression variables x and y respectively. Although this approximation can affect drastically their dynamics, later on in the decay phase, it will not change the return to equilibrium of the firing rate h. In Fig. D , we compare system (1) (continuous line) and (2) (dashed line) for 3 different values of synaptic weight J (not too large): while the depression and facilitation variable are much affected, the firing rate dynamics is pretty robust.

Analysis of the approximated system

Because in system (2) the dynamics of x and y do not depend anymore on h, we can now integrate them and obtain

$$\begin{aligned}
h(t) &= H \exp\left(-\frac{t}{\tau} + J\int_0^t x(s)y(s)ds\right) \\
x(t) &= A + B\exp(-\alpha t) \\
y(t) &= \exp\left(-f(t)\right)\left(1 + \frac{1}{t_d}\int_0^t \exp(f(s))ds\right),
\end{aligned} \tag{3}$$

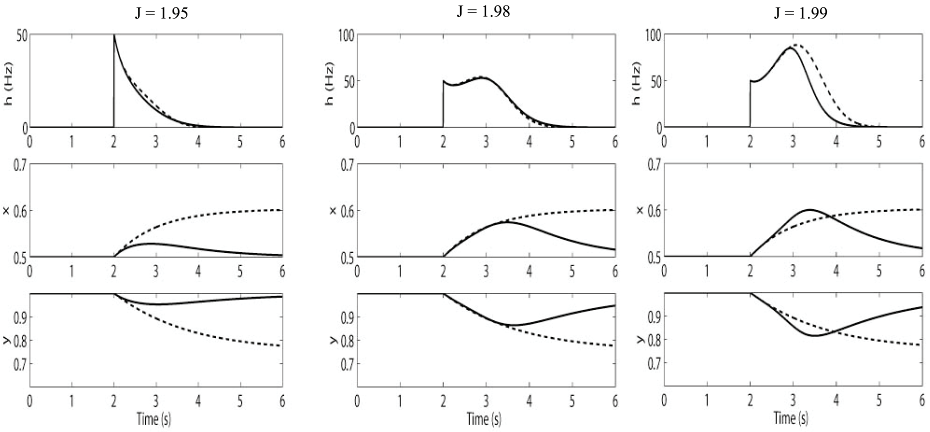

Figure D. Comparison of system of equations (1) **(continuous line) and the approximated system** (2) **(dashed line)**. We use three different values of the connectivity parameter J. The firing rate h, the facilitation x and the depression y variables are plotted as functions of time. For a low enough connectivity parameter J, the firing rate is well approximated.

where

$$A = X\left(1 + \frac{KH}{\alpha}\right), B = -X\left(\frac{KH}{\alpha}\right), \alpha = \frac{1}{t_f} + KH \tag{4}$$

and

$$f(t) = \frac{t}{t_d} + LH\int_0^t x(s)ds, \text{ with } f(0) = 0 \text{ and } f'(0) = \frac{1}{t_d} + LHX. \tag{5}$$

Because the function f increases with the time, we further approximate

$$\int_0^t \exp(f(s))ds \approx \int_0^t \exp(f(t) + f'(t)(s-t))ds \tag{6}$$

$$\approx \frac{\exp(f(t))(1 - \exp(f'(t)))}{f'(t)} \tag{7}$$

leading with equation 3 to

$$y(t) = \exp(-f(t)) + \frac{1 - \exp\left(-\frac{t}{t_d} - tLHx(t)\right)}{1 + t_d LHx(t)}. \tag{8}$$

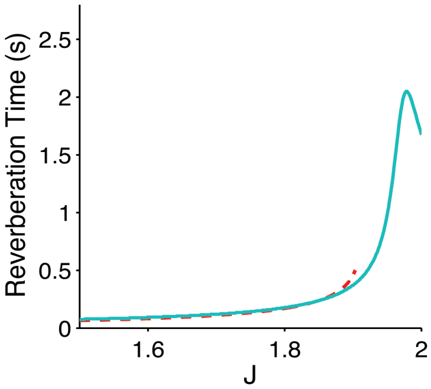

Figure E. Comparison between the depression variable y estimated by equation (8) (blue) and the exact one obtained by numerical simulation of system (2) (black).

This approximation is quite robust as demonstrate by figure E. Finally, using expressions 3 and 8, we obtain for the firing rate h

$$h(t) \quad = \quad H \exp\left(-\frac{t}{\tau} + J\int_0^t x(s)y(s)ds\right). \tag{9}$$

Approximation of the firing rate h

To obtain an explicit expression for the firing rate variable h, we now decompose the last term into two parts

$$\int_0^t x(s)y(s)ds = \overbrace{\int_0^t x(s)\exp(-f(s))ds}^{\text{I}} + \overbrace{\int_0^t x(s)\frac{1-\exp\left(-\frac{s}{t_d} - sLHx(s)\right)}{1+t_dLHx(s)}ds}^{\text{II}}. \tag{10}$$

The first term I is

$$I = A\int_0^t \exp(-f(s))ds + B\int_0^t \exp(-\alpha t - f(s))ds. \tag{11}$$

Because the term I is the sum of two integrals of decreasing functions, we use Laplace's method at the point 0, which is a regular. Thus using relations

4

$$
\begin{aligned}
A\int_0^t \exp(-f(s))ds &\approx A\int_0^t \exp(-f(0)-sf'(0))ds \approx A\frac{\exp(-f(0))(\exp(-tf'(0))-1)}{-f'(0)}\\
&\approx \frac{A}{\frac{1}{t_d}+LHX}\left[1-\exp\left(t\left(\frac{1}{t_d}+LHX\right)\right)\right].
\end{aligned}
$$

Furthermore,

$$
\begin{aligned}
B\int_0^t \exp(-\alpha t-f(s))ds &\approx B\int_0^t \exp(-f(0)-s(\alpha+f'(0)))ds \qquad (12)\\
&\approx \frac{B}{\alpha+\frac{1}{t_d}+LHX}\left[1-\exp\left(t\left(\alpha+\frac{1}{t_d}+LHX\right)\right)\right].
\end{aligned}
$$

Finally,

$$
I \approx AF_\beta(t)+BF_{\alpha+\beta}(t), \qquad (13)
$$

where $\beta=\frac{1}{t_d}+LHX$, $F_0(t)=t$ and

$$
F_u(t)=\frac{1-e^{-ut}}{u}, \quad t\in\mathbb{R}, u\in\mathbb{R}^*. \qquad (14)
$$

We shall now estimate II using that $t_dLH=0.54<1$ and $x(t)<1$. Expanding in Taylor series

$$
(1+t_dLHx(t))^{-1}=\sum_{k=0}^{\infty}(-t_dLHx(t))^k, \qquad (15)
$$

yields

$$
\begin{aligned}
II &= \int_0^t x(s)(1-\exp(-sf'(s)))\sum_{k=0}^{\infty}(-t_dLHx(s))^k ds\\
&= \sum_{k=0}^{\infty}(-t_dLH)^k\int_0^t x(s)^{k+1}(1-\exp(-sf'(s)))ds\\
&= \sum_{k=0}^{\infty}(-t_dLH)^k\int_0^t\sum_{i=0}^{k+1}\binom{k+1}{i}A^iB^{k+1-i}e^{-\alpha s(k+1-i)}(1-e^{-sf'(s)})ds,
\end{aligned}
$$

where we have used equation 3. Finally, we obtain that

$$
\begin{aligned}
II &\approx \sum_{k=0}^{\infty}(-t_d LH)^k \sum_{i=0}^{k+1}\binom{k+1}{i} A^i B^{k+1-i}(F_{\alpha(k+1-i)}(t) - F_{\beta+\alpha(k+1-i)}(t)) \qquad (16)\\
&\approx \sum_{k=0}^{\infty}(-t_d LH)^k A^{k+1} \sum_{i=0}^{k+1}\left(\frac{B}{A}\right)^i \binom{k+1}{i}(F_{\alpha i}(t) - F_{\beta+\alpha i}(t)), \qquad (17)
\end{aligned}
$$

where

$$
|\frac{B}{A}| = \frac{\frac{KH}{\alpha}}{(1+\frac{KH}{\alpha})} < 1. \qquad (18)
$$

Summarizing the previous estimates, a power series expansion in the variable $-t_d LH$ for

$$
\begin{aligned}
\int_0^t x(s)y(s)ds &\approx AF_\beta(t) + BF_{\alpha+\beta}(t) \qquad (19)\\
&+ \sum_{k=0}^{\infty}(-t_d LH)^k A^{k+1} \sum_{i=0}^{k+1}\left(\frac{B}{A}\right)^i \binom{k+1}{i}(F_{\alpha i}(t) - F_{\beta+\alpha i}(t))\\
&\approx At + BF_\alpha(t) + \sum_{k=1}^{\infty}(-t_d LH)^k A^{k+1} \sum_{i=0}^{k+1}\left(\frac{B}{A}\right)^i \binom{k+1}{i}(F_{\alpha i}(t) - F_{\beta+\alpha i}(t))\\
&\approx At + BF_\alpha(t) + A\sum_{k=1}^{\infty} C^k \sum_{i=0}^{k+1}\left(\frac{B}{A}\right)^i \binom{k+1}{i}(F_{\alpha i}(t) - F_{\beta+\alpha i}(t)), \qquad (20)
\end{aligned}
$$

where $C = -t_d LHA$. Reorganizing the series by changing the order of summation, we get

$$
\begin{aligned}
\int_0^t x(s)y(s)ds &\approx At + BF_\alpha(t) + A\sum_{i=1}^{\infty}\sum_{k=i}^{\infty} C^k \left(\frac{B}{A}\right)^i \binom{k+1}{i}(F_{\alpha i}(t) - F_{\beta+\alpha i}(t))\\
&+ B\sum_{i=1}^{\infty}(BC)^i(F_{\alpha(i+1)}(t) - F_{\beta+\alpha(i+1)}(t)) + A(t - F_\beta(t))\sum_{i=1}^{\infty} C^i \qquad (21)
\end{aligned}
$$

Using the value of the parameters, the variable B and $\frac{B}{A}$ are small and Thus, we shall neglect terms of order greater than 2 in order of B and $\frac{B}{A}$ to obtain

$$\begin{aligned}\int_0^t x(s)y(s)ds &\approx At + BF_\alpha(t) + B\sum_{k=1}^{\infty}(k+1)C^k(F_\alpha(t) - F_{\beta+\alpha}(t)) + \frac{AC}{1-C}(t - F_\beta(t)) \\ &\approx At + BF_\alpha(t) + B\left(\frac{1}{(1-C)^2} - 1\right)(F_\alpha(t) - F_{\beta+\alpha}(t)) + \frac{AC}{1-C}(t - F_\beta(t)).\end{aligned}$$

Finally using equation 9, we obtain an approximated expression for the rate h

$$h(t) \approx H\exp\left\{\frac{-t}{\tau} + J\left[\frac{At}{1-C} + \frac{BF_\alpha(t)}{(1-C)^2} - \frac{AC}{1-C}F_\beta(t) - B\left(\frac{1}{(1-C)^2} - 1\right)F_{\alpha+\beta}(t)\right]\right\}. \quad (22)$$

The reverberation time satisfies a transcendental equation

We derive here a transcendental for the reverberation time T_R. For that purpose, we follow the experimental protocol where an induced spike (at time zero) sets the firing rate to a value H. The reverberation time T_R is then defined as the first time where the firing rate reaches the threshold h_{th} that is

$$T_R = \inf\{t > 0,\, h(t) = h_{th}\}. \quad (23)$$

We can now use expression 22 to estimate the reverberation T_R as a function of the synaptic and network parameters:

$$\begin{aligned}h_{th} = h(T_R) &\approx H\exp\left\{\frac{-T_R}{\tau} + J\left[\frac{AT_R}{1-C} + \frac{BF_\alpha(T_R)}{(1-C)^2} - \frac{AC}{1-C}F_\beta(T_R)\right.\right. \\ &\left.\left. -B\left(\frac{1}{(1-C)^2} - 1\right)F_{\alpha+\beta}(T_R)\right]\right\}. \quad (24)\end{aligned}$$

At this stage, we conclude that the reverberation time T_R is solution of a transcendental equation

$$\begin{aligned}\ln\left(\frac{H}{h_{th}}\right) &= \left(\frac{1}{\tau} - \frac{JA}{1-C}\right)T_R - J\left[\frac{BF_\alpha(T_R)}{(1-C)^2} - \frac{AC}{1-C}F_\beta(T_R)\right. \\ &\left. -B\left(\frac{1}{(1-C)^2} - 1\right)F_{\alpha+\beta}(T_R)\right]. \quad (25)\end{aligned}$$

Analytical approximation of the reverberation time

To obtain an explicit expression for the reverberation time T_R as function of J, we shall expand the exponential terms in the transcendental equation, which can be written as

$$J = \Phi(T_R) = \frac{T_R - T_0}{\tau G(T_R)}, \tag{26}$$

where

$$\begin{aligned} T_0 &= \tau \ln\left(\frac{H}{h_{th}}\right) & (27)\\ G(t) &= \frac{A}{1-C}t + \frac{BF_\alpha(t)}{(1-C)^2} - \frac{AC}{1-C}F_\beta(t) - B\left(\frac{1}{(1-C)^2} - 1\right)F_{\alpha+\beta}(t). \end{aligned}$$

When the reverberation time T_R is short enough, we can Taylor expand the function G to second order polynomial, denoted by $P(t)$, where

$$\begin{aligned} P(t) &= \frac{A}{1-C}t + \frac{B(t-\frac{\alpha}{2}t^2)}{(1-C)^2} - \frac{AC}{1-C}(t - \frac{\beta t^2}{2}) - \frac{BC(2-C)}{(1-C)^2}(t - \frac{(\alpha+\beta)t^2}{2})\\ &= (A+B)t + \frac{-\alpha B + \beta\left(AC(1-C) + B\left(1-(1-C)^2\right)\right)}{2(1-C)^2}t^2\\ &= (A+B)t + \frac{1}{2}\left(-\alpha B + \frac{\beta\left(-(A+B)C^2 + (A+2B)C\right)}{(1-C)^2}\right)t^2. & (28) \end{aligned}$$

Using $A + B = X$ and $\alpha B = -XKH$, we obtain that

$$P(t) = Xt + \frac{1}{2}\left(XKH + \frac{\beta C\left(-XC + X + B\right)}{(1-C)^2}\right)t^2. \tag{29}$$

With $a = t_f KH$ and $b = t_d LHX$, we have $B = -\dfrac{Xa}{1+a}$ and $C = -b\left(1 + \dfrac{a}{1+a}\right)$. Thus, we obtain that

$$P(t) = Xt + \frac{X}{2}\left(\frac{a}{t_f} - \frac{b(b+1)\left(1+\frac{a}{1+a}\right)\left(b\left(1+\frac{a}{(1+a)}\right)+\frac{1}{1+a}\right)}{t_d(1+b\left(1+\frac{a}{1+a}\right))^2}\right)t^2 \tag{30}$$

a and b are small parameters. A final expansion of P in third order in the parameters a and b yields

$$P(t) = Xt + \frac{X}{2}\left(\frac{a}{t_f} - \frac{b}{t_d}\right) t^2 \tag{31}$$

$$= Xt + \frac{XH}{2}(K - LX)\, t^2. \tag{32}$$

Using this equation to solve (26), we are left with solving

$$J\tau P(T_R) - T_R + T_0 = 0$$

$$\Leftrightarrow \quad \frac{J\tau XH\,(K - LX)}{2} T_R^2 + (XJ\tau - 1)T_R + T_0 = 0. \tag{33}$$

Retaining the solution that satisfies $T_R = T_0 = \tau \ln\left(\frac{H}{h_{thresh}}\right)$ for $J = 0$, we finally get

$$T_R(J) = \frac{1 - JX\tau - \sqrt{(J\tau X - 1)^2 - 2J\tau XH(K - LX)\tau \ln\left(\frac{H}{h_{th}}\right)}}{J\tau XH(K - LX)}. \tag{34}$$

In figure E, we compare this expression with the exact solution: we obtain a good agreement for $J < 1.9$. When $J > 1.9$, the reverberation time is larger than $1s$. Thus, the approximation made in equation (28) is not valid anymore. However, it is possible to approximate T_R linearly for $J \in [1.9; 1.99]$. Using the implicit function theorem. We can locally invert equation (26) to obtain

$$T_R(J) \approx 1 + \frac{\tau G^2(1)}{G(1) - (1 - T_0)G'(1)}(J - \Phi(1)) \tag{35}$$

With the parameters (table 1), we obtain that

$$T_R(J) \approx 25.38J - 48.36. \tag{36}$$

We have obtained here precise estimates for the reverberation time, in the range $J \in [0; 1.99]$. This range includes the value $J = 1.98$, used in the model. Two regimes have to be considered defined by the synaptic parameter J. When the network is not sufficiently connected, the reverberation T_R is slowly increasing as a function of J (equation (34)). For a network sufficiently connected ($J > 1.9$) T_R becomes linear. In figure F,we plotted these estimates, which show good agreement with numerical simulations. Finally, for larger values of J, an analytical expression of T_R as a function of J remains to be found.

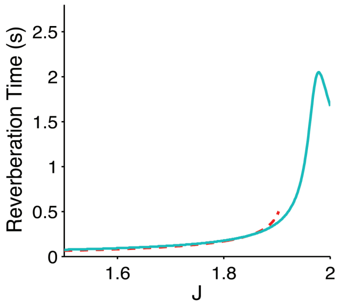

Figure F. Comparison between numerical simulations and estimates of the reverberation time T_R. The reverberation time is plotted as a function of J for the exact model (solid line), the approximated model (dash black line), and the estimates given by equations (34) (dash red line) and (36) (blue dash line).

References

[1] Cohen D, Segal, M, (2011) Network bursts in hippocampal microcultures are terminated by exhaustion of vesicle pools. *J Neurophysiol* 106(5):2314-21
